# A live-cell image-based machine learning strategy for reducing variability in PSC differentiation systems

**DOI:** 10.1038/s41421-023-00543-1

**Published:** 2023-06-06

**Authors:** Xiaochun Yang, Daichao Chen, Qiushi Sun, Yao Wang, Yu Xia, Jinyu Yang, Chang Lin, Xin Dang, Zimu Cen, Dongdong Liang, Rong Wei, Ze Xu, Guangyin Xi, Gang Xue, Can Ye, Li-Peng Wang, Peng Zou, Shi-Qiang Wang, Pablo Rivera-Fuentes, Salome Püntener, Zhixing Chen, Yi Liu, Jue Zhang, Yang Zhao

**Affiliations:** 1 State Key Laboratory of Natural and Biomimetic Drugs, MOE Key Laboratory of Cell Proliferation and Differentiation, Beijing Key Laboratory of Cardiometabolic Molecular Medicine, Institute of Molecular Medicine, College of Future Technology, Peking University, Beijing, China; 2 Academy for Advanced Interdisciplinary Studies, Peking University, Beijing, China; 3 Beijing Key Lab of Traffic Data Analysis and Mining, School of Computer and Information Technology, Beijing Jiaotong University, Beijing, China; 4 College of Engineering, Peking University, Beijing, China; 5 College of Chemistry and Molecular Engineering, Synthetic and Functional Biomolecules Center, Beijing National Laboratory for Molecular Sciences, Key Laboratory of Bioorganic Chemistry and Molecular Engineering of Ministry of Education, Peking University, Beijing, China; 6 State Key Laboratory of Membrane Biology, College of Life Sciences, Peking University, Beijing, China; 7 Peking-Tsinghua Center for Life Sciences, Peking University, Beijing, China; 8 Department of Chemistry, University of Zurich, Zurich, Switzerland; 9 Institute of Chemical Sciences and Engineering, Ecole Polytechnique Fédéral de Lausanne, Lausanne, Switzerland; 10 Institute of Molecular Medicine, National Biomedical Imaging Center, Beijing Key Laboratory of Cardiometabolic Molecular Medicine, College of Future Technology, Peking University, Beijing, China

**Keywords:** Stem-cell differentiation, Pluripotent stem cells, Biological techniques

## Abstract

The differentiation of pluripotent stem cells (PSCs) into diverse functional cell types provides a promising solution to support drug discovery, disease modeling, and regenerative medicine. However, functional cell differentiation is currently limited by the substantial line-to-line and batch-to-batch variabilities, which severely impede the progress of scientific research and the manufacturing of cell products. For instance, PSC-to-cardiomyocyte (CM) differentiation is vulnerable to inappropriate doses of CHIR99021 (CHIR) that are applied in the initial stage of mesoderm differentiation. Here, by harnessing live-cell bright-field imaging and machine learning (ML), we realize real-time cell recognition in the entire differentiation process, e.g., CMs, cardiac progenitor cells (CPCs), PSC clones, and even misdifferentiated cells. This enables non-invasive prediction of differentiation efficiency, purification of ML-recognized CMs and CPCs for reducing cell contamination, early assessment of the CHIR dose for correcting the misdifferentiation trajectory, and evaluation of initial PSC colonies for controlling the start point of differentiation, all of which provide a more invulnerable differentiation method with resistance to variability. Moreover, with the established ML models as a readout for the chemical screen, we identify a CDK8 inhibitor that can further improve the cell resistance to the overdose of CHIR. Together, this study indicates that artificial intelligence is able to guide and iteratively optimize PSC differentiation to achieve consistently high efficiency across cell lines and batches, providing a better understanding and rational modulation of the differentiation process for functional cell manufacturing in biomedical applications.

## Introduction

Pluripotent stem cell (PSC)-derived differentiated functional cells in theory provide an unlimited cell source for regenerative medicine, in vitro modeling of biological development and diseases, and drug screening and evaluation^1–3^. However, one of the major problems of current PSC differentiation is the line-to-line and batch-to-batch variabilities^4–8^ because cells are likely inclined to misdifferentiation trajectory. The variability of the PSC differentiation leads to repeated experiments, making the derivation of functional cells time- and labor-intensive. In addition, the evaluations of differentiation results usually rely on low-throughput or destructive methods (e.g., immunofluorescence), hampering quality control during differentiation and downstream applications. All of these severely impede the progress of scientific research and manufacturing of cell products. The line-to-line variability is mostly driven by the PSC genetic and epigenetic variations which could change the pluripotency network or the signaling response of developmental pathways, leading to differentiation capacity bias for specific lineages^9–12^. Other inevitable non-genetic variations in routine cell culturing, like cell passage number^13^ and altered handling of cells among laboratories or individuals^14–18^, also contribute to the differentiation variability. Moreover, since the PSC differentiation is a stepwise process including multiple induction stages, small perturbations or inconsistency at early stages would accumulate and magnify^6^, intensifying the differentiation vulnerability. Therefore, monitoring and intervening in the overall differentiation process non-invasively is necessary for consistently high-efficiency PSC differentiation.

At present, the variability of the PSC differentiation could be partially controlled by individual experience. Based on the observation of cell images, the experimenter empirically modulates the experiment scheme in time and prejudges the differentiation outcome^19–22^. However, these experiences on cell images are varying and difficult to quantify, replicate and impart; moreover, rapid or subtle changes in cell images are hard to be captured by experimenters.

**Fig. 1:**
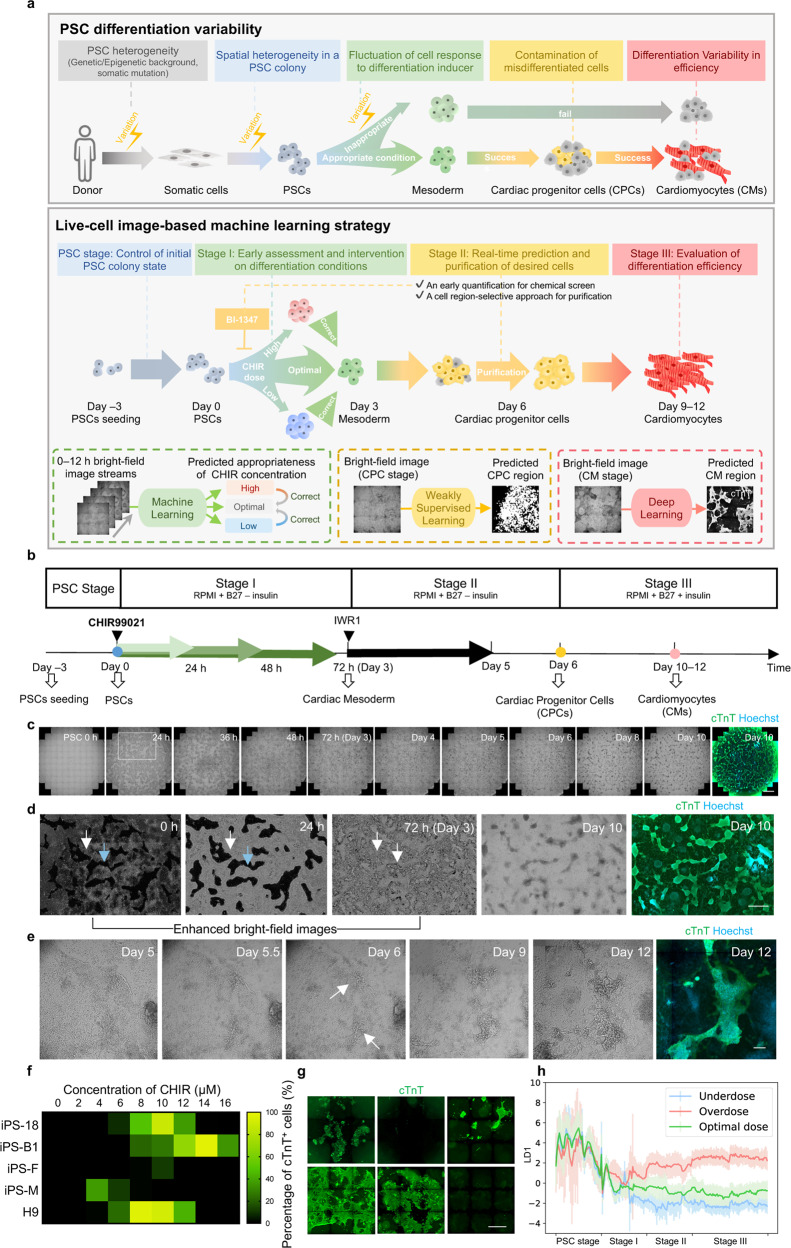
Framework for image analysis on the PSC-to-CM differentiation process. **a** Schematic overview of the PSC differentiation strategy based on image-based ML for solving the variability in efficiency, using CM differentiation as an example. Upper panel, variation occurs at each step in the PSC differentiation process. Lower panel, based on the bright-field image-based ML, our strategy is applicable at different stages to reduce the variation, achieving high-efficiency CM induction. **b** Schematic overview of PSC-to-CM differentiation with small-molecule modulators of canonical Wnt signaling. The green arrows (indicating the titration of CHIR durations and concentrations at stage I) and the colored dots represent the checkpoints involving ML in our strategy. **c** Example time-lapse bright-field images and cTnT fluorescence result of cardiac differentiation for 10 days. Cells were cultured in a 24-well plate. The enlargement of the image in the white frame is shown in **d**. Scale bar, 1 mm. **d** Location and morphology of successfully and unsuccessfully differentiated cells in the whole differentiation process. 0 h, 24 h, and 72 h bright-field images were enhanced and shown in inverted color for highlighting the edge of the cell colony (white arrows and blue arrows indicate the cell-containing area and cell-free area, respectively). Scale bar, 1 mm. **e** Texture and morphological change of successfully differentiated cells from day 5 to day 12. The white arrow indicates the day 6 CPC with texture. Scale bar, 250 μm. **f** Line-to-line variability of cardiac differentiation efficiency. CHIR duration = 24 h. **g** Batch-to-batch variability of cardiac differentiation efficiency. iPS18-derived CM differentiation followed the same differentiation protocol but from different batches for 12 days (CHIR concentration = 6 μM, duration = 24 h). Scale bar, 1 mm. **h** Change of image local features under different CHIR doses in cardiac differentiation. The *y*-axis is the first linear discriminant (LD1) given by LDA. The dark lines and the light lines represent the mean and the standard deviation value, respectively.

Currently, state-of-the-art microscopic technologies could support long-term, time-lapse, high-throughput image acquisition on live cells. Meanwhile, the fast-evolving machine learning (ML) method is being increasingly applied in cellular image analysis, which is opening up possibilities to recognize specific cellular constituents or cell lineages during differentiation in cell culture^23–31^. During PSC differentiation, cell fate transitions involve rapid changing of cell morphology and arrangement^32–36^. Thus, we hypothesized that microscopic images of unlabeled cells contained enough information of the differentiation state which could be captured by ML. This information could be used for interventions in the differentiation process to correct the cell trajectory in time and eliminate misdifferentiated cell contamination. In this study, based on live-cell bright-field images, we developed a strategy harnessing different ML models, which can identify cell lineage non-invasively, modulate the differentiation process in real-time, and optimize the differentiation protocol, improving the invulnerability in PSC-to-functional cell differentiation (Fig. 1a).

## Results

### Image trajectory analysis for the PSC-to-cardiomyocyte differentiation process

To explore the feasibility of modulating the PSC-based differentiation process via bright-field images, we first chose the differentiation process of cardiomyocyte (CM) as an example case because CM is in urgent need for cardiovascular disease study and regenerative medicine^1,37^. The time-lapse bright-field image information was first collected and analyzed throughout the human PSC-to-CM differentiation process following a chemical-defined PSC differentiation protocol (Fig. 1b; Supplementary Fig. S1a–d)^20^. Briefly, the activation of Wnt signaling (CHIR99012, CHIR) can specifically induce PSCs to mesoderm (stage I, day 0–3), and the application of Wnt signaling inhibitor (IWR1) induces the cells to cardiac progenitor cells (CPC stage, stage II, day 4–6). Then CPC converts to CM after the addition of insulin (CM stage, stage III, day 7–~12). And in this process, CHIR concentrations and durations were key determinants for CM differentiation efficiency^20,38^.

We induced PSC-to-CM following this well-established protocol^20^ and recorded the time-lapse bright-field images (Fig. 1b–e; Supplementary Video S1). For increasing the diversity of image information, we intentionally introduced several variables (e.g., different human PSC lines, initial cell confluency, differentiation medium, etc.), and in particular titrated CHIR doses, namely CHIR concentrations (ranging from 2 μM to 14 μM) and durations (ranging from 24 h to 48 h) for each batch, covering underdose, optimal dose, and overdose conditions. Throughout the ~15 days differentiation process (day –3 to ~12), cells in each well were imaged ~250 times, forming time-lapse bright-field image streams. In total, 1152 image streams were collected including more than 7,200,000 snaps. At the end of the differentiation, the successfully differentiated CMs were recognized by immunostaining of cTnT, a CM-specific marker, and fluorescent imaging. The total fluorescence intensity of cTnT, termed the “Differentiation Efficiency Index”, was used to quantify the efficiency. Image streams for wells with different efficiency were collected, due to the intrinsic viability in the differentiation process and aforementioned variables. Consistent with previous studies^20^, we found that the optimal CHIR concentration differs among cell lines (Fig. 1f), and even for a certain cell line with a given condition, the differentiation efficiency and cell morphology could vary among batches, as indicated by cTnT immunostaining (Fig. 1g), although parallel wells under the same CHIR dose within one batch remained consistent.

We next investigated whether bright-field images could informatively indicate different differentiation status. By extracting 448-dimensional local features (SIFT^39^, SURF^40^, and ORB^41^) from the whole-well bright-field images in the PSC-to-CM differentiation process, we found that the local image features distributed differently among cells with high and low differentiation efficiencies, and also among cells with different differentiation stages, as shown by Principal Component Analysis (PCA) score plots (Supplementary Fig. S2a, b). Linear discriminant analysis (LDA)^42^ of local features indicated that the image trajectory gradually diverged over time among different CHIR doses (Fig. 1h; Supplementary Fig. S2c). These findings support that the bright-field image streams contained informative clues reflecting the PSC differentiation stage, differentiation efficiency, and CHIR dose.

### Deep learning-based CM recognition and efficiency evaluation

**Fig. 2:**
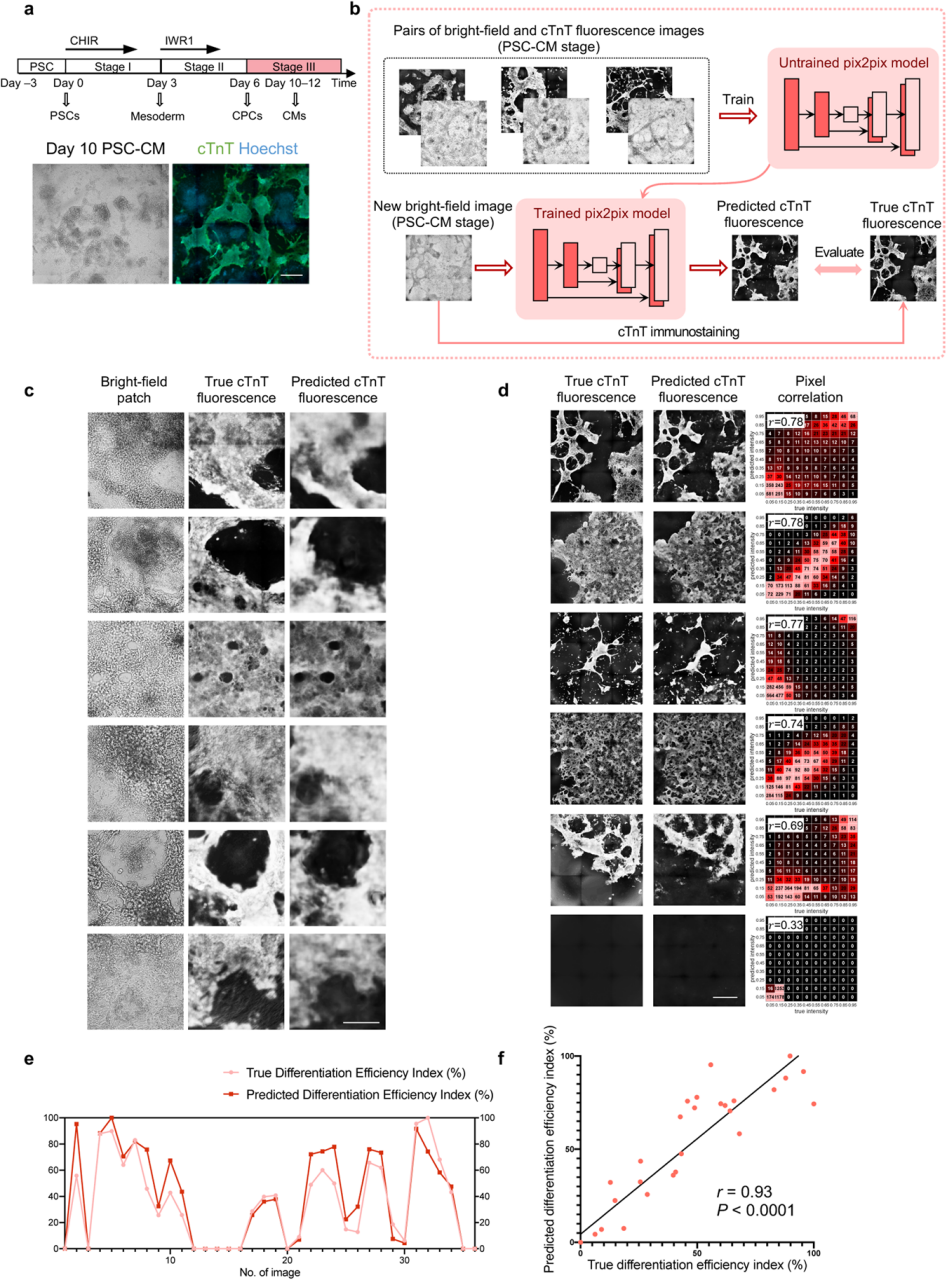
CM recognition and differentiation efficiency evaluation by deep learning on bright-field images. **a** Morphological characteristics of CMs and non-CMs on day 12 bright-field images and cTnT fluorescence results. Scale bar, 500 μm. **b** Schematic overview of deep learning-based cTnT fluorescence prediction from live-cell bright-field images at CM stage (stage III). The pix2pix model was trained with pairs of bright-field and fluorescence images. The trained model could predict the fluorescent labels for new bright-field images. For evaluation, the model’s prediction was further compared with the true cTnT fluorescence image. **c** Typical predicted results of CM recognition on bright-field image patches (containing cTnT^+^ CMs) from the test set. Each column from left to right represents: live-cell bright-field image patches; true cTnT fluorescence results; predicted cTnT fluorescence results. Scale bar, 250 μm. **d** Typical predicted results of CM recognition on whole-well bright-field images. Each column from left to right: true cTnT fluorescence result; predicted cTnT fluorescence result; the heatmap comparing the predicted with the true fluorescence pixel intensities, with Pearson’s *r* values provided. Scale bar, 1 mm. **e** Comparison of the true and predicted Differentiation Efficiency Indexes (i.e., total fluorescence intensity) for whole-well fluorescence images. True and predicted Differentiation Efficiency Indexes were normalized between 0 and 100%. *n* = 36 wells. **f** Correlation between the true and predicted Differentiation Efficiency Indexes, with Pearson’s *r* value. True and predicted Differentiation Efficiency Indexes were normalized between 0 and 100%. *n* = 36 wells.

We next characterized the local features of the various cell lineages in the bright-field images in greater detail. Upon intensively examining the live-cell bright-field images for each well, we noticed that the successfully differentiated cTnT^+^ CMs exhibited characteristic features, including a compact, domed, 3D morphology. Moreover, the successfully differentiated CMs were typically connected in sheets or cords (Fig. 2a). The morphology of cells that did not differentiate into CMs was heterogeneous without an obvious pattern of aggregation. These findings support the feasibility of using bright-field images to recognize CMs, potentially based on local features per se.

Pursuing this, we used deep convolutional neural networks (CNNs) to recognize CMs from live-cell bright-field images by predicting the cTnT fluorescent labels. The pix2pix model^43^ was adopted for the bright-field-to-fluorescence image transformation task (Fig. 2b; Supplementary Fig. S3a–c). Through end-to-end training with paired bright-field and true fluorescence images, the model can capture the multi-scale features of CMs, which enables it to generate fluorescence predictions for new bright-field images.

We prepared a dataset of paired bright-field images and true (i.e., obtained experimentally) fluorescence images for each well, with various differentiation efficiencies and different cell lines included to increase its diversity (Supplementary Table S1). On the test set, the predicted cTnT fluorescence intensities matched with the true ones at the pixel level, suggesting that our model could accurately recognize the CMs (Fig. 2c, d; Supplementary Fig. S4a, b). As for the whole-well differentiation efficiency, the Pearson correlation value between the predicted and the true Differentiation Efficiency Index reached *r* = 0.93 (*P* < 0.0001; Fig. 2e, f); and the trained model could also recognize CMs on a new dataset from three additional cell lines, with a Pearson correlation value *r* = 0.81 (*P* < 0.0001; Supplementary Fig. S4c, d). Together, we achieved non-invasive recognition of PSC-derived functional cells from bright-field images and evaluation of differentiation efficiency.

### Weakly supervised learning-based CM-committed CPC recognition and efficiency prediction

**Fig. 3:**
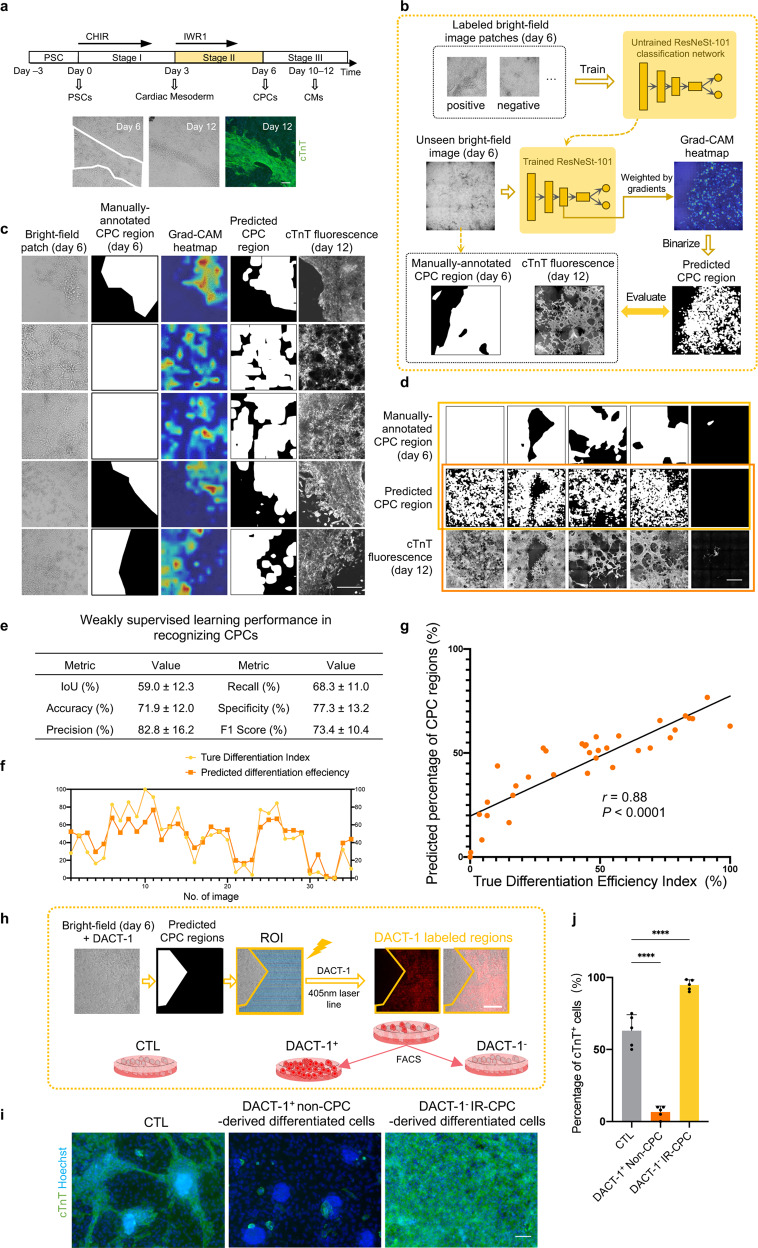
CM-committed CPC recognition and purification by weakly supervised learning on bright-field images. **a** Morphological characteristics of CPCs (circled by white lines) and non-CPCs on day 6. Representative day 6 bright-field image, day 12 bright-field image, and cTnT fluorescence results were shown. Scale bar, 200 μm. **b** Schematic of the weakly supervised learning model for CPC recognition from day 6 live-cell bright-field images. The ResNeSt network was trained with bright-field patches labeled with “positive” or “negative”. For a new bright-field image, the trained ResNeSt combined with Grad-CAM could generate a heatmap highlighting the regions important to the ResNeSt’s inference. The prediction of CPC regions was obtained by binarizing the heatmap. For evaluation, the predicted CPC regions were compared with manually annotated CPC regions (segmentation masks) on day 6 and the final cTnT fluorescence image at the CM stage. **c** Typical predicted results of CPC recognition on positive bright-field patches from the test set. Each column from left to right represents: live-cell bright-field image patches on day 6; manually annotated CPC regions; Grad-CAM heatmap for CPC localization; binary prediction of CPC regions; true cTnT fluorescence result of day 12. Scale bar, 250 μm. **d** Typical predicted results of CPC recognition on whole-well bright-field images from the test set. Each row from top to bottom represents: manually annotated CPC regions; binary prediction of CPC regions; true cTnT fluorescence result of day 12. Statistical analysis was performed on the first two rows (in the yellow frame) shown in **e** and the last two rows (in the red frame) shown in **f**, **g**. Scale bar, 1 mm. **e** Performance of CPC recognition, evaluated by comparing manually annotated CPC regions and binary prediction of CPC regions. Data are means ± SD. *n* = 35 wells. **f** Comparison of the true Differentiation Efficiency Index (from day 12 cTnT fluorescence results) and the predicted percentage of CPC region (from day 6 bright-field images). The true Differentiation Efficiency Index was normalized between 0 and 100%. *n* = 35 wells. **g** Correlation between the true Differentiation Efficiency Index (from day 12 cTnT fluorescence results) and the predicted percentage of CPC regions (from day 6 bright-field images), with Pearson’s *r* value. The true Differentiation Efficiency Index was normalized between 0 and 100%. *n* = 35 wells. **h** Experimental setup of DACT-1-based CPC purification. Cells were preincubated by DACT-1 on day 6; cells were selectively photoactivated (405 nm laser) using ROI scanning mode of a microscope guided by the predicted result; then the fluorescence-labeled cells (λ_max_ = 560 nm) and unlabeled cells were digested into single cells and separated by FACS to achieve purification. Scale bar, 100 μm. **i** Purification effect of day 6 IR-CPCs. After FACS, DACT-1-labeled non-CPCs, unlabeled IR-CPCs, and cells of the control (CTL) group were subsequently cultured for 6 days in RPMI + B27 medium. The purity of subsequently differentiated CMs was identified by immunostaining of cTnT. All the cells were from the same batch and under the same differentiation condition. Scale bar, 100 μm. **j** Quantitative analysis of the percentage of cTnT^+^ cells in **i**. Data are means ± SD. *n* = 5 images. *P < 0.05; ****P < 0.0001 by one-way ANOVA followed by Dunnett’s multiple comparisons tests.

The success of CM recognition demonstrated that the successfully differentiated cells contained distinguishable features, which encouraged us to study the image features of cells in the CPC stage. By observing the bright-field image stream, we found that CM-committed CPCs of day 6 were a group of more stereoscopic spindle-like cells with stronger contrast (Figs. 1e, 3a; Supplementary Videos S1, S2). This suggested that it was possible to apply ML to recognize CM-committed CPCs based on the day 6 bright-field images. Although some biomarkers for CM-committed CPCs^44–46^ have been reported, using one or two biomarkers was not exclusive enough for precise labeling. Those CPCs which would yield cTnT^+^ CMs subsequently could be directly tracked by image streams. Considering that CPC proliferation and migration resulted in a mismatch of the day 6 CPC regions and the day 12 cTnT^+^ regions, we explored using weakly supervised learning for recognizing CM-committed CPC based solely on image-level categorical labels^47,48^ (i.e., without the pixel-level labels used to train the pix2pix model for CM recognition detailed above).

We used a two-stage design in our weakly supervised learning model for recognition (Fig. 3b; Supplementary Fig. S5a, b). First, a CNN-based classification network (specified as ResNeSt-101^49^) was trained with 8463 bright-field image patches from day 6, labeled as positive (≥ 30% CM-committed CPC regions) or negative (Supplementary Fig. S6a, b and Table S2; Materials and methods). The CPC regions were manually annotated by tracking the location of cTnT^+^ cells in the image streams (from day 12 back to day 6). Next, the bright-field images from the test set were passed to the trained network, and Gradient-weighted Class Activation Mapping (Grad-CAM)^50^ was used to generate heatmaps that could highlight the regions contributing most to the network’s inference. The prediction of CPC regions was therefore derived from the highlighted regions in the heatmaps.

We first evaluated the model performance on the test set by comparing the predicted CPC regions with manually annotated CPC regions. The prediction of the CM-committed CPC regions from day 6 bright-field images was similar to the annotated regions, with an accuracy of 71.9 ± 12.0% (Fig. 3c–e; Supplementary Fig. S6c). As for the performance in predicting final differentiation efficiency in advance, the predicted percentage of CM-committed CPC regions (day 6) displayed a linear correlation with the true Differentiation Efficiency Index computed from the cTnT fluorescence labels (day 12) (Pearson’s *r* = 0.88, *P* < 0.0001) (Fig. 3f, g), regardless of the cell proliferation and migration in a whole well. The prediction result of CM-committed CPCs on a new dataset from three additional cell lines also had a significant linear correlation with the true Differentiation Efficiency Index (Pearson’s *r* = 0.83, *P* < 0.0001) (Supplementary Fig. S6d, e). Collectively, through weakly supervised learning, CM-committed CPCs were recognized non-invasively and thus the cardiac differentiation efficiency could be predicted at an early stage. These provided an opportunity for intervention in the progenitor cell stage, such as early termination of experiment with low efficiency or purification of experiment with relatively high efficiency. We named these groups of CM-committed CPCs identified from bright-field images as image-recognized CPCs (IR-CPCs).

### Region-selective purification and characterization of image-recognized CPCs

Since CPCs could be recognized from bright-field images by weakly supervised learning, we attempted to purify those IR-CPCs for reducing cell contamination without cell surface markers, which is different from traditional purification methods^44,45^. We adopted a non-cytotoxic photoactivatable probe, Dual-Activatable Cell Tracker 1 (DACT-1)^51^, which can be activated relying on both intracellular carboxylesterases and photoirradiation (λ = 405 nm), and its fluorescent photoproduct could retain inside the irradiated cells, to realize cell labeling in assigned region (Fig. 3h; Supplementary Fig. S7a). After that, on-demand cells could be isolated from other cells by fluorescence-activated cell sorting (FACS).

Following the aforementioned purification workflow, IR-CPC regions on day 6 were assigned and misdifferentiated cells (non-CPCs) were irradiated. After being sorted by FACS and cultured in the regular medium of the CM stage, IR-CPCs yielded the cTnT^+^ CMs at 94.70 ± 3.70% in comparison to 6.60 ± 4.22% of the non-CPCs and 63.00 ± 11.16% of unsorted day 6 cells (Fig. 3i, j). In addition, while irradiating IR-CPCs instead of non-CPCs, it could also achieve obviously higher purity compared with unsorted cells but possibly cause cell injury (indicated by lower beating frequency) due to the laser phototoxicity (Supplementary Fig. S7b, c). Likewise, the same approach was effective on image-recognized CMs (IR-CMs) purification on day 12 (Supplementary Fig. S7d, e). Thus, based on CM or CPC recognition model, we presented an on-demand cell sectioning and purification approach by region-selective photoactivation, realizing the high purity of terminally differentiated cells.

Immunofluorescence analysis of the IR-CPCs showed co-expression of classical markers of CPC (NKX2.5, GATA4, MEF2C, ISL1)^52^; notably, some cells without the IR-CPC texture also expressed some of these markers (Supplementary Fig. S8a, b). An RNA-seq analysis also revealed that purified IR-CPCs had gene expression signatures similar to those reported during the progenitor stage of cardiac differentiation in the literature (*NKX2–5*, *GATA4*, *MEF2C*, *TBX5*, *TBX20*, *ISL1*, *HAND1*, *HAND2*)^53^ (Supplementary Fig. S9a–f). CMs showed an 8.98-fold higher expression of *TNNI3*, a CM marker gene, compared with IR-CPCs (*P* < 0.05), and other CM marker genes (*TNNT2, TNNC1*, *MYH6*, *MYH7*) had consistent trends. In addition, non-CPCs expressed genes characteristic of primary epicardial cells (EP) (*WT1* and *TBX18*), of fibroblasts (*COL1A1*, *COL1A2*, *VIM*, *BMP1*), and of endothelial cells (EC) (*PECAM1*, *CDH5*, *KDR*) (Supplementary Fig. S9c). IR-CPCs possessed the molecular features of CPCs in heart development, indicating the fidelity of image-based CPC recognition and purification.

### ML-based early assessment of CHIR doses

**Fig. 4:**
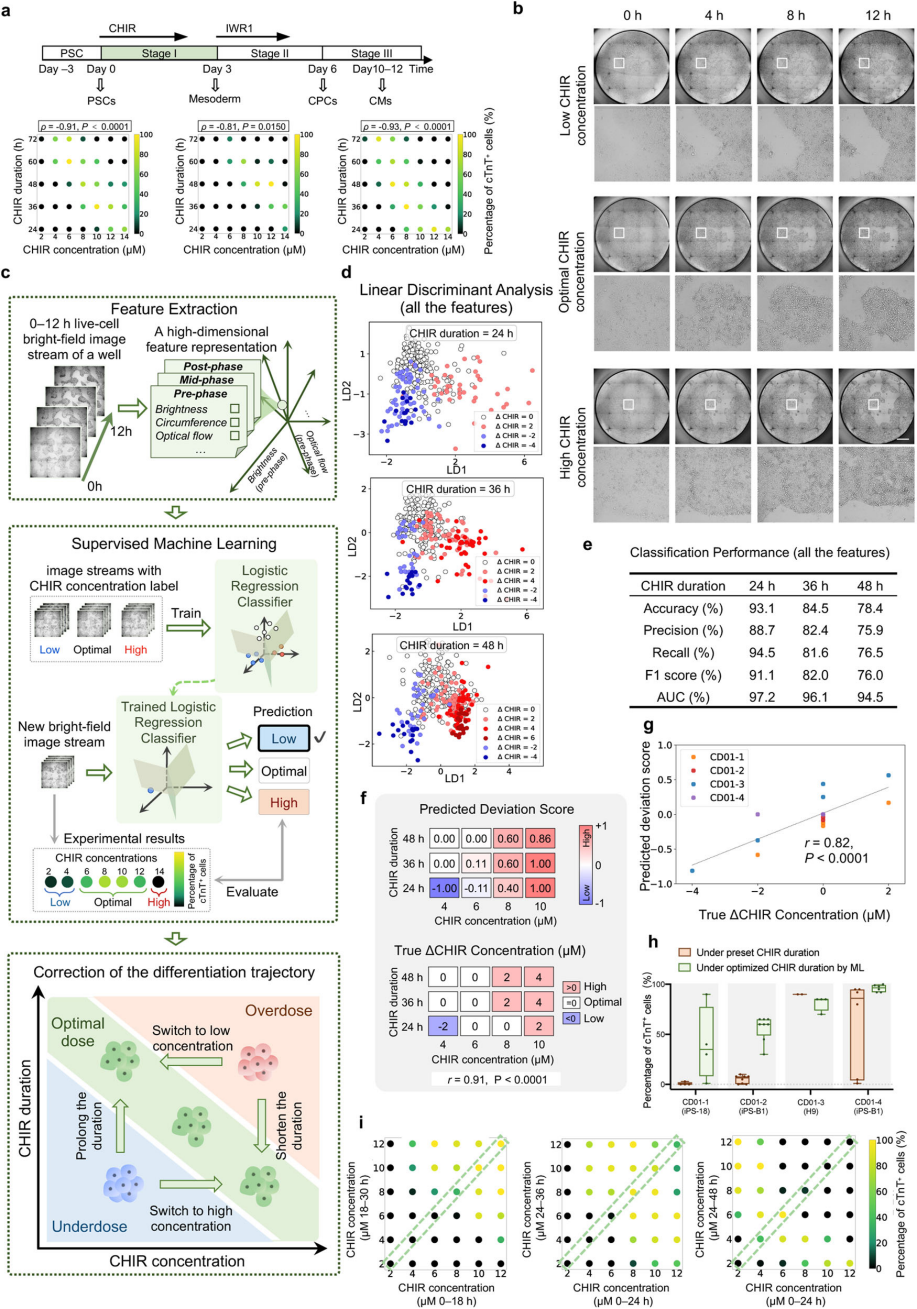
Early assessment and adjustment of CHIR dose by supervised ML on 0–12 h bright-field image streams. **a** Titration of CHIR concentration and duration for three batches. Each dot’s color represents the mean percentage of cTnT^+^ cells on day 12 (*n* = 3 wells for each condition). Correlation analysis was conducted between CHIR concentrations and durations for high-efficiency wells (percentage of cTnT^+^ cells ≥ 50%), with Spearman’s *ρ* value. **b** Typical bright-field image streams under different CHIR concentrations from 0 h to 12 h. A partially enlarged view is provided below each image. Scale bar, 1 mm. **c** Schematic overview of the CHIR dose assessment and adjustment by supervised ML on 0–12 h live-cell bright-field image streams. Upper panel: each whole-well image stream was represented by a 21-D feature. Middle panel: In the training stage, the logistic regression model was trained with pairs of bright-field image streams and their CHIR concentration label; in the testing stage, the trained model made predictions of CHIR concentration (low, optimal, high) for new bright-field images. Lower panel: misdifferentiated cells can be rescued by further intervention. **d** LDA on 0–12 h image streams represented by 21-D features. LDA linearly projects the 21-D features into a 2-D plane spanned by the most discriminant axes (denoted by LD1 and LD2). *n* = 384 wells. **e** Classification performance of the logistic regression model (using all the features) on the test set. Precision, recall, F1 score, and AUC were macro-averaged over the three categories. *n* = 116 wells. **f** Comparison of predicted Deviation Scores (upper panel) and true ΔCHIR Concentrations (lower panel) for each CHIR dose condition in a testing batch, with Pearson’s *r* value. *n* = 12 CHIR doses. **g** Classification performance of cross-batch validation with Pearson’s *r* value (under a CHIR duration of 24 h). The color of the dots represents different testing batches. *n* = 20 CHIR doses. **h** The effects of adjusting CHIR duration based on ML prediction in different cell lines and batches. The percentage of cTnT^+^ cells for wells under a preset CHIR condition (8 μM, 48 h) and under an optimized condition (8 μM, ML-selected duration) were shown. *n* = 2–6 wells. Statistical significance was determined by *t*-test, ***P < 0.001; ****P < 0.0001. **i** Effect of CHIR concentration switching on differentiation efficiency for three batches. In the middle of the CHIR treatment, the initial CHIR concentrations (shown on the *x*-axis) were switched to other concentrations (shown on the *y*-axis).

As abovementioned, the cell recognition and purification in CM and CPC stages (stage III and II) have solved the problem of misdifferentiated cell contamination, while we expected those misdifferentiated cells could be corrected to the right trajectory at an early stage (Stage I) of differentiation based on bright-field images. The inducer we used in stage I (CHIR) was known as a determinative inducer cardiac differentiation^5,20,38^. However, the optimum dose range of CHIR was very limited, cell line-dependent, and always variable among batches^30^ (Figs. 1f, g, 4a). This variable cell response to CHIR might result from the different signaling basis of cell lines^9^, variation of albumin^54^, and different PSC cell-cycle profiles with varying culture confluency^5^, accounting for the instability of cardiac differentiation across batches^30^. Intriguingly, during titrating CHIR concentrations and durations within one batch, we found a negative correlation between CHIR concentration and duration of high-efficiency wells (Spearman correlation value *ρ* = –0.80 ± 0.14, *n* = 8 batches) (Fig. 4a). This provided a rule for flexible adjustment of CHIR concentration or duration to achieve high efficiency if inappropriate CHIR doses could be assessed in time.

Therefore, we tested whether the CHIR doses for each batch could be assessed based on the very early bright-field images. Through detailed observations of the changes of images during 0–12 h CHIR treatment, we found that the PSC colonies shrunk, becoming denser and more obvious (Fig. 4b), which might contain potentially useful features about the actual response to CHIR. We then explored supervised ML to classify the wells into low, optimal, and high CHIR concentration groups under a selected CHIR duration, expecting ML could learn to capture the relationship between image stream features and the appropriateness of CHIR doses. We built a logistic regression model upon handcrafted features of 0–12 h image streams (Fig. 4c). The dataset for training and testing the model consisted of image streams from different cell lines, batches, CHIR doses, etc (Supplementary Table S3). Each image stream was assigned to a CHIR concentration group (low, optimal, high) according to the final percentage of cTnT^+^ cells (Materials and methods). Inspired by previous observations, we designed 21 features (at three phases, each with seven features) (Fig. 4c; Supplementary Fig. S10a), and these features were adequate to distinguish the wells in different CHIR concentration groups as shown by the LDA score plot (Fig. 4d).

The logistic regression model was trained and tested for three selected CHIR durations (24 h, 36 h, 48 h) separately. On the test set, the logistic regression model reached an accuracy of 93.1%, 84.5%, and 78.4% when the CHIR durations were selected, respectively, as 24 h, 36 h, and 48 h (Fig. 4e). We also trained and tested the model using the four most discriminative features (selected by ANOVA), which were post-phase Optical Flow^55^, post-phase and mid-phase Cell Brightness, and post-phase Colony Circumference (Supplementary Fig. S10b). The model still preserved good discriminative ability and the feature redundancy was reduced as shown by PCA, LDA, and the classification performance (Supplementary Fig. S10c–f). These data indicated that supervised ML could assess the CHIR doses only by relying on 0–12 h bright-field images.

### Early correction of the differentiation trajectory by adjusting the CHIR dose

As our model could accurately assess the CHIR concentration in a very early stage (~12 h), we next investigated whether the assessment from ML could guide the adjustment of CHIR dose in time for high efficiency. We first defined a “Deviation Score” as the model prediction about the appropriateness of CHIR concentration under a selected CHIR duration. For comparison, “ΔCHIR Concentration” was introduced to reflect the true deviation from the optimal CHIR concentration derived from experimental results (Materials and methods). The predicted Deviation Score and the true ΔCHIR Concentration shared a similar trend, with a Pearson correlation value *r* = 0.91 (*P* < 0.0001; Fig. 4f). The result of cross-batch validation also supported this conclusion, with a Pearson correlation value *r* = 0.82 (*P* < 0.0001; Fig. 4g; Supplementary Fig. S10g, h), which indicated that our model was still valid on new batches.

Using the predicted Deviation Score as a reference, we could optimally adjust the CHIR dose by either changing the CHIR duration or concentration in practice. With the model’s prediction (i.e., Deviation Scores) under different selected CHIR durations (24 h, 36 h, or 48 h), the optimal CHIR duration (which had the Deviation Score closest to 0) could be obtained at ~12 h (Fig. 4f; Supplementary Fig. S10a). The feasibility of adjusting CHIR duration for higher efficiency had been verified in the experiment (Fig. 4a, h). In addition, we could also switch the CHIR concentration according to the model’s prediction. We verified that switching CHIR concentration in the middle of CHIR treatment could induce CMs with higher efficiency, balancing the previous treatment of inappropriate CHIR concentrations (Fig. 4i; Supplementary Fig. S10i). Together, we realized timely intervention on the condition of a determinative differentiation inducer by image-based ML, promisingly stabilizing the differentiation with high efficiency.

### Controlling the initial state of PSC colonies

**Fig. 5:**
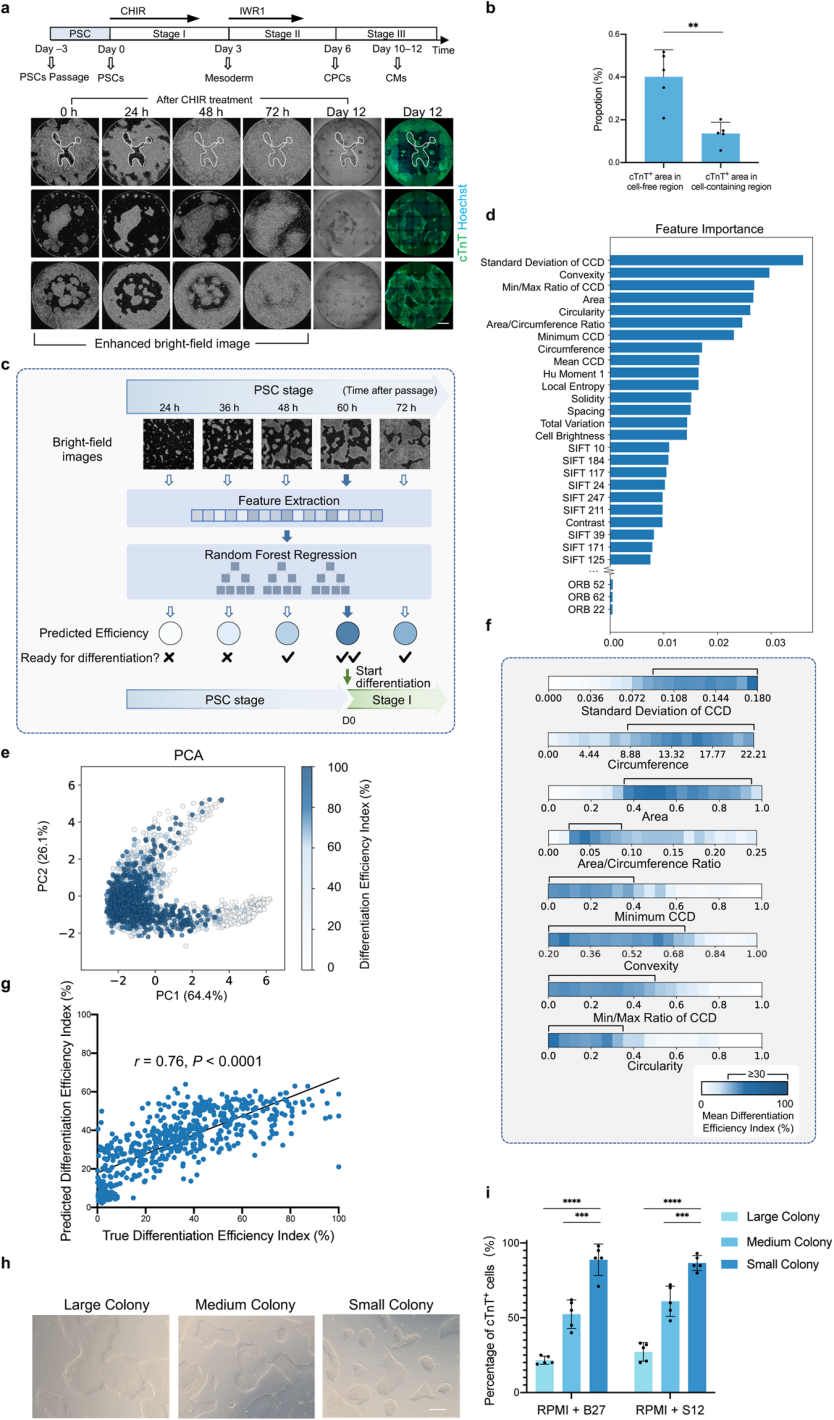
Controlling the initial state of PSC colonies hinted by time-lapse images. **a** Different differentiation tendencies between cells located at the periphery and center (spatial heterogeneity) of the PSC colony indicated by the image streams. The 0–72 h bright-field images were enhanced and shown in inverted color for a clearer colony edge. After CHIR treatment, PSC colonies start to contrast at 0 h; starting from ~24–48 h, cells located at the periphery of the colony migrate into the cell-free region (circled in time-lapse images of the first row as an example) until the whole well was filled at ~72 h. This group of migrating cells is likely to successfully differentiate, while the cells located at the center of a large colony tend to fail. Scale bar, 1 mm. **b** Comparisons between the proportion of the cTnT^+^ area (day 12) in cell-free regions and in cell-containing regions (0 h). Data are means ± SD. *n* = 5 wells. Statistical significance was determined by paired *t*-test, **P < 0.01. **c** Schematic diagram of the ML-based control of initial PSC colony states. During the PSC stage, image features of PSC colonies were extracted and passed to a random forest regression model for efficiency prediction in real-time, providing guidance for identifying the most conducive starting point for differentiation. **d** Importance weights of the 343 features determined by the random forest model. The feature importance weights were computed on the training set (*n* = 1350 wells). CCD, Centroid-Contour Distance. **e** PCA plot of the eight features with the most importance weights in **d**. The color represented the Differentiation Efficiency Index, which was normalized between 0 and 100%. *n* = 1934 wells. **f** The relationships between the mean differentiation efficiency and the eight features with the most importance weights shown in **d**. The range of each feature was divided into 20 bins, and the mean Differentiation Efficiency Index in each bin was computed. *n* = 1934 wells. **g** Correlation between the true and the predicted Differentiation Efficiency Index, with Pearson’s *r* value. *n* = 584 wells. **h** Bright-field images (0 h, before CHIR treatment) of PSC colonies with different sizes, controlled by digestion time and operation during passaging. The number of initial PSCs was strictly identical among wells. Scale bar, 50 μm. **i** Effect of different PSC colony sizes on differentiation efficiency using RPMI + B27 protocol and RPMI + S12 protocol. Data are means ± SD. *n* = 5. Statistical significance was determined by *t*-test, ***P < 0.001; ****P < 0.0001.

We found that there still remained cells failing to differentiate to CMs even under the optimal CHIR dose, which motivated us to investigate the relation between differentiating cell heterogeneity and CM induction capability within a well (Fig. 5a). Tracking image streams in reverse order, back to the PSC stage, we found that cells migrated from the periphery of the initial PSC colonies beginning at 24 h (after differentiation was initiated), and almost filled the cell-free area by 72 h (Fig. 5a; Supplementary Videos S1, S3), eventually yielding CMs, whereas cells located in the center of large PSC colonies more frequently failed to fully differentiate. Quantitative image analysis revealed that the proportion of cTnT^+^ areas (day 12) in cell-free regions (0 h) was significantly higher than in cell-containing regions, confirming our observations (Fig. 5b). The trend of spatially variable differentiation within PSC colonies led us to hypothesize that colony morphology could contribute to the differentiation process. We therefore built a model to determine the optimal PSC colony shape that led to the high differentiation efficiency (Fig. 5c).

To this end, we induced differentiation of various PSC colonies from different cell lines at a range of times after passage (Supplementary Table S4) and quantified their morphological profiles at 0 h (before CHIR treatment) by 343 features of the bright-field images. For each batch, the final cTnT fluorescence images were collected and only wells under the optimal CHIR condition were considered. Random forest modeling revealed that Standard Deviation, Minimum, and Min/Max Ratio of Centroid-Contour Distances, as well as Colony Area, Circumference, Area/Circumference Ratio, Circularity, and Convexity, were the features most relevant to high-efficiency cell differentiation (Fig. 5d, e). The relationships between each individual feature and final efficiency further implied that initial colonies with moderate area and with longer, irregular peripheries tended to have higher differentiation efficiency (Fig. 5f), which was consistent with our observations. Using this morphology-based random forest regression model, we found that PSC differentiation efficiency under the optimal CHIR condition could be predicted based on the PSC morphological profiles at 0 h with a high correlation (Pearson’s *r* = 0.76, *P* < 0.0001) (Fig. 5g), which enabled us to monitor PSC colonies after passage in real-time by ML to identify the most conducive starting point for differentiation.

In addition, since the colony periphery was also affected by passaging operations, we further extended the periphery length and reduced the central area by enzymatically digesting larger initial colonies into smaller ones while passaging (Fig. 5h). The changes in passaging operations to reduce colony size resulted in a PSC-to-CM differentiation efficiency of 88.8% ± 10.5%, compared to 21.6% ± 2.7% for larger colonies that started with the same confluence of initial PSCs (Fig. 5i). This non-invasive ML-based assessment of colony morphology combined with optimization of the passaging protocols together resulted in flexible and reproducible control of differentiation efficiency through modulation of the initial PSC colony state.

### Small molecule screening based on the CPC-recognition model to improve the resistance to the overdose of CHIR

Since we had modulated the cardiac differentiation process by ML, we further investigated whether the differentiation protocol per se could be optimized. Given that CHIR concentration fluctuation is known to account for much of the instability of cardiac differentiation (Figs. 1f, g, 4a)^5,20,38^, increasing the cell resistance to inappropriate CHIR concentrations for differentiation is another solution for improving the reproducibility of the cardiac differentiation. The gene expression profiles under different CHIR doses at stage I (PSCs to cardiac mesoderm, day 0–3) were characterized by bulk RNA-seq. Presomitic mesoderm master genes were upregulated in CHIR overdose groups, consistent with previous reports^38,56,57^, while cells treated with optimum CHIR doses possessed a cardiac mesoderm gene expression signature (Supplementary Fig. S11a–d). Thus, we attempted to maintain cardiac lineage by counteracting the differentiation tendency to presomitic mesoderm by small molecule screening.

**Fig. 6:**
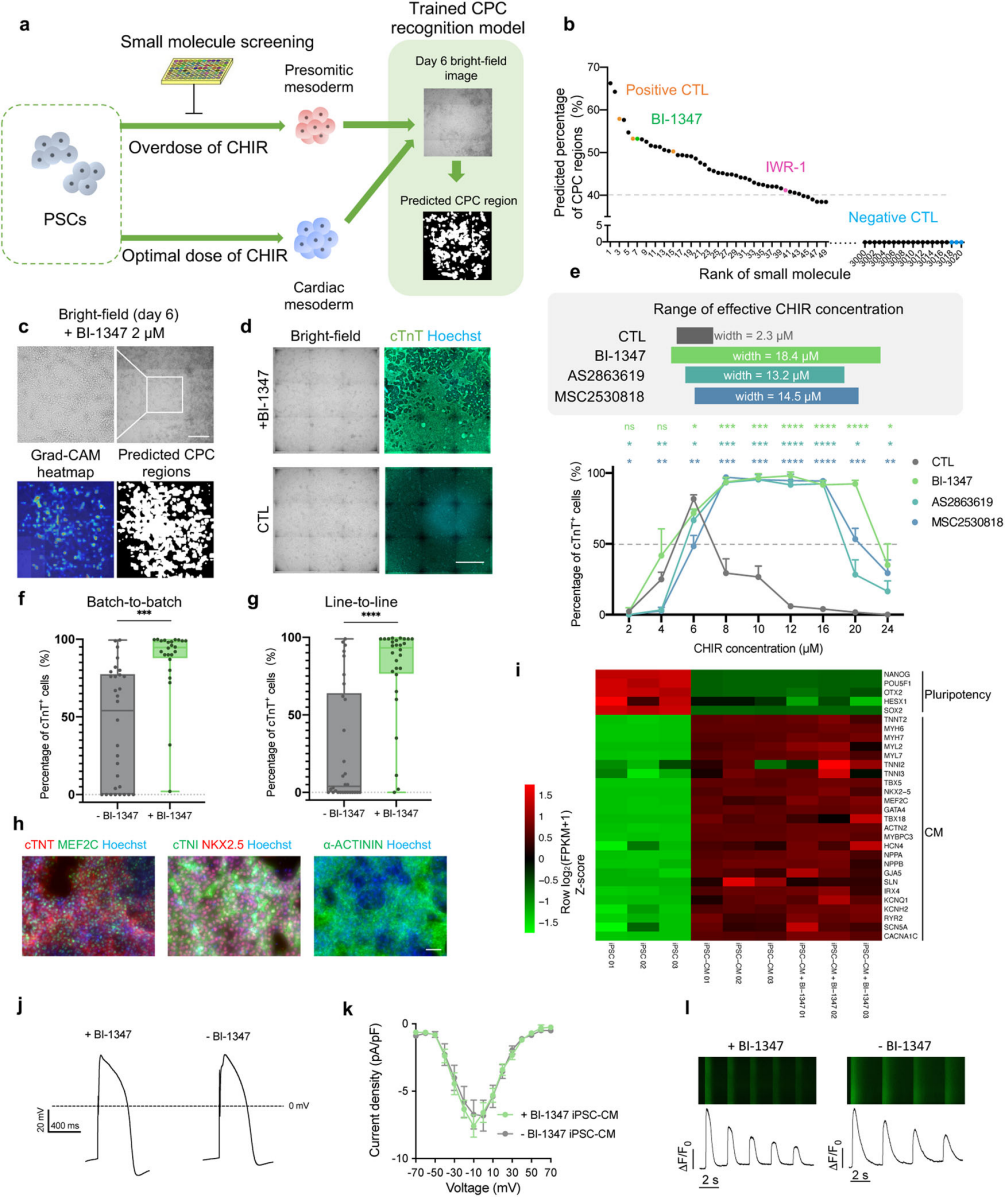
Small molecule screening for broadening the range of effective CHIR concentration based on the CPC recognition model. **a** Schematic overview of the small molecule screening. A ~3000-compound library was screened for counteracting the differentiation tendency to presomitic mesoderm under a high CHIR dose (16 μM, 0–48 h). The differentiation efficiencies were estimated by using the CPC recognition model on day 6 bright-field images. **b** Preliminary small molecule screening result ordered by the predicted percentage of CPC regions on day 6. Cells of positive CTL were treated with optimal CHIR, while cells of negative CTL (DMSO) or library screening groups were treated with overdose CHIR (16 μM, 48 h). **c** Effect of BI-1347 (2 μM, 48 h) during preliminary screening. The heatmap and the predicted CPC regions are generated by the CPC recognition model. Scale bar, 500 μm. **d** Effect of BI-1347 on improving differentiation efficiency under high CHIR dose (20 μM, 48 h). Representative bright-field images and fluorescence images of cTnT on day 12 were shown. Scale bar, 1 mm. **e** Effect of CDK8 inhibitors (0.5 μM, 48 h) on improving the differentiation efficiency under titration of CHIR concentrations using iPS-B1 line. CHIR was added with BI-1347, AS2863619, or MSC2530818 for 0–48 h. The widths of effective CHIR concentration (percentage of cTnT^+^ cells ≥ 50%, indicates by a dashed line) were shown in the upper panel. Data are means ± SD. *n* = 3. Statistical significance was determined by *t*-test to compare the BI-1347, AS2863619, and MSC2530818 groups with the CTL group under each CHIR concentration. *P < 0.05, **P < 0.01, ***P < 0.001, ****P < 0.0001; ns, not significant. **f** Effect of BI-1347 on improving the consistency of batch-to-batch differentiation efficiencies. Cells from different batches were treated with CHIR (6 μM, 48 h) or CHIR (12 μM, 48 h) plus BI-1347 (0.5 μM, 48 h) on the iPS-B1 line. ***P < 0.001. **g** Effect of BI-1347 on improving the consistency of line-to-line differentiation efficiencies. Cells from six different lines (iPS-B1, iPS-18, iPS-F, iPS-M, H9, WIBR3) were treated with CHIR (6 μM, 48 h) or CHIR (12 μM, 48 h) and BI-1347 (0.5 μM, 48 h). ****P < 0.0001. **h** Immunofluorescent result of CM with BI-1347 treatment (0.5 μM, 48 h) under high CHIR dose (16 μM, 48 h). Representative fluorescent images of CM with BI-1347 treatment for cTnT, MEF2C, cTNI, NKX2.5, and α-ACTININ on day 12 were shown. Scale bar, 100 μm. **i** Gene expression data for iPSC, iPSC-CM without BI-1347 treatment, and with BI-1347 (0.5 μM, 48 h)- treated iPSC-CM (day 12, both without metabolic purification). Heatmap shows Z-scores of log_2_(FPKM + 1) normalized over multiple samples. **j** Action potential in iPSC-CMs with (+BI-1347, 0.5 μmol/L, 0–48 h) or without (–BI-1347) BI-1347. **k** I–V plots of *I*_*Ca*_ in iPSC-CMs with or without BI-1347. Data are expressed as means ± SEM. *n* = 15–16. **l** Fluorescent images (upper) and time courses (lower) of spontaneous calcium transients in iPSC-CMs with or without BI-1347.

Considering that a CPC recognition model had been constructed for predicting differentiation efficiency in advance, we established a screening platform using day 6 bright-field images as an early output, instead of identifying CMs (Fig. 6a; Supplementary Fig. S12a). PSCs were exposed to each of ~3000 small molecules (from the commercial library and in-house libraries) with CHIR of high concentration (16 μM) during 0–48 h. The top 40 compounds were considered as initial candidates, which could sufficiently promote the cardiac lineage commitment with the predicted percentage of CM-committed CPC regions ≥ 40% (Fig. 6b). Given that the effective CHIR concentration for cardiac differentiation was restricted to a narrow range, all the initial candidates were subsequently tested under different CHIR concentrations (CHIR duration = 48 h) for the ability to maintain cardiac lineage.

After effect verification of the initial candidates, BI-1347 (a CDK8 selective inhibitor), was confirmed to have robust and stable effects on broadening the range of effective CHIR concentration (mean 6.4-fold in different PSC lines), realizing reproducibly high efficiency of cardiac differentiation of different batches or lines (Fig. 6c–g; Supplementary Fig. S12b, c). BI-1347 functioned in a dose-dependent manner after testing, with an optimal concentration range from 0.05 to 0.5 μM (Supplementary Fig. S12d, e). The qualities of CMs with or without BI-1347 treatment were basically identical, as indicated by their morphology, contractile properties, and expression of CM-specific genes (Fig. 6h–l; Supplementary Fig. S13a–i and Videos S4, S5). Two additional CDK8 selective inhibitors (AS2863619, MSC2530818) exhibited similar effects to BI-1347 (Fig. 6e).

As previously reported, CDK8 functioned as a transcriptional repressor or activator^58^, and also interacted with the Wnt pathway in cancer models^59,60^. Therefore, we wondered whether the Wnt inhibitor had a similar effect to BI-1347. After adding IWR1 (a Wnt inhibitor), the range of effective CHIR concentration was not markedly broadened compared with BI-1347 (Supplementary Fig. S14a). This indicated that inhibiting CDK8 activity could safeguard cardiac lineage differentiation with higher reproducibility and CDK8 inhibitors were not simply functioned via Wnt pathway. By observing the bright-field images, BI-1347 might work via preventing cell death confirmed by propidium iodide (PI) staining at 48 h (Supplementary Fig. S14b–d), which might not fully account for the promotion of CM differentiation efficiency using BI-1347. To explore the dramatic enhancement of cardiomyocyte induction by CDK8 inhibitor, we applied bulk RNA-seq and found that under the treatment of overdose CHIR, BI-1347 blocked the fate of presomitic mesoderm^38,56,57^, and led to consistent cell differentiation into cardiac mesoderm by resisting CHIR overdose (Supplementary Fig. S14e–g). Together, these results presented a promising capacity of our CPC recognition model in offering a high-throughput screening platform with an early output, and further optimizing the cardiac differentiation method by improving its invulnerability.

### Applying the image-based strategy to nephric and hepatic differentiation

Our proof-of-concept studies in image-based ML-guided cardiac differentiation encouraged us to transfer our strategy to modulate other PSC differentiation processes, such as PSC differentiation into nephric cells and hepatic cells, which are also valuable for cell-based therapy or drug toxicity evaluation. In the early differentiation process of PSCs to kidney organoids^19^, the optimal CHIR concentration was essential to the high differentiation efficiency but fluctuated among batches, depending on the cell line, the passage number, and the culture conditions^61,62^ (Supplementary Fig. S15a). However, cells under treatment of different CHIR concentrations (low, optimal, high) exhibited distinct bright-field image characteristics (loose, normal, and dense, respectively) on day 4 (when CHIR was removed)^19^ (Supplementary Fig. S15b). We then tested whether CHIR concentration could be assessed on day 4 by ML.

We prepared a dataset of day 4 bright-field images with different cell lines (iPS-B1, iPS-F, iPS-M, H9, WIBR3) and CHIR concentrations (ranging from 3 μM to 16 μM). For assessing CHIR concentration, bright-field images on day 4 were labeled as low, optimal, or high according to their characteristics on day 4 and SIX2 (a marker for nephron progenitor cells, NPCs) immunofluorescent staining on day 9 (Supplementary Fig. S15c). The t-SNE plot of SIFT local features extracted from bright-field images indicated clear separation among different CHIR concentration groups (Supplementary Fig. S15d). Using these local features, a trained logistic regression model could accurately classify the bright-field images in the test set with an accuracy of 99.11% (Supplementary Fig. S15e, f). Since the cells displaying dense morphology should be terminated early, and the cells displaying loose morphology could be corrected by prolonging the CHIR treatment for high-efficiency differentiation^19^, the early assessment of CHIR concentration provided us with valuable guidance for stabilizing a nephric differentiation system.

Low reproducibility in differentiation efficiency across batches is also a critical challenge for hepatic differentiation systems^63–67^. We therefore explored the application of ML for non-invasively recognizing definitive endoderm (DE) regions (72 h, stage I of hepatic differentiation) in bright-field images for early assessment of hepatic differentiation status and subsequent potential image-based cell purification, as described above (Supplementary Fig. S16a). Live-cell bright-field images and their SOX17 (a DE marker gene) immunofluorescent images were captured at 72 h, which involved varying differentiation efficiency by modulating the dose of small molecules (CHIR and IDE1) used in stage I of differentiation in different cell lines (Supplementary Fig. S16b). We then trained the weakly supervised learning model on DE stage bright-field images with purely categorical labels (i.e., “positive” or “negative”, according to the proportion of SOX17^+^ cell region, see Materials and methods). After training, the endoderm cell regions predicted by the trained model matched well with the SOX17 fluorescence labels (Supplementary Fig. S16c). The proportion of predicted endoderm cell regions was also correlated with the proportion of true SOX17^+^ cell regions (Pearson’s *r* = 0.92, *P* < 0.0001) (Supplementary Fig. S16d). These two extended applications further validated the generalizability of our strategy.

## Discussion

We here developed a general PSC differentiation strategy to intelligently modulate and optimize the differentiation process based on label-free image-based ML, thus greatly reducing the vulnerability of differentiation (Fig. 1a). Throughout the cardiac differentiation process, our strategy integrated control of the initial PSC state, early assessment and intervention in differentiation conditions (e.g., CHIR dose), purification of the desired cells recognized by ML, and protocol optimization by ML-assisted small molecule screening, together providing a solution for consistently high-quality differentiation from PSCs to functional cells. Our robust strategy can boost cell manufacturing for biomedical applications and provides a new perspective for understanding the biology of PSC differentiation.

Distinct from some applications of ML for biological tasks^25,29^, the informative image features we observed and applied for PSC quality control and CHIR dose assessment have plausible biological explanations. Consistent with our finding that cells located at the periphery or the center of colonies have exhibited different differentiation tendencies, there are previous reports of spatial heterogeneity of signaling responses within an PSC colony^68–70^. Similar to our observation of CM-committed cells migrating to the cell-free space, the formation of the mesoderm is known to require cell migration through the primitive streak during embryogenesis^71,72^. Among the most discriminative image features applied to CHIR assessment in cardiac differentiation, Optical Flow, for tracking cell movements, can reflect that cells undergo epithelial-mesenchymal transition for mesoderm formation^73^. And Colony Circumference and Cell Brightness can measure the colony size and colony density, which have been reported to affect the CHIR response of cells: the Wnt signaling was limited to the periphery of high-density colonies which was mediated by E-cadherin, while Wnt signaling was modestly biased to the periphery in low-density colonies^68^. These image characteristics in our strategy are informative for analyzing differentiation processes in biological systems.

Our strategy can be generalized for use in other PSC differentiation processes (e.g., the nephric differentiation presented above). PSC heterogeneity and instability of differentiation conditions (e.g., CHIR doses) are common to the differentiation process of PSCs into other functional cells, which impedes large-scale cell production. Since the microscopic images of cells contain potentially informative signals regarding cell lineage (e.g., morphology and aggregation dynamics)^32–36,74^, our image-based strategy should be applicable to PSC differentiation into other cell types, for instance monitoring the rosette formation in PSC-to-neuron differentiation process^34,75^. More broadly, adopting our strategy may be useful for cell biotechnologies beyond 2D PSC differentiation, such as organoids differentiation^76–79^, somatic reprogramming to PSC^80^, or direct cell lineage reprogramming^81^.

Notably, prior knowledge from researches about differentiation provides valuable guidance for strategy development. Observation of cell morphology and knowledge about the determinative inducer can direct the design of imaging, the condition of inducer treatment, and ML algorithms. While choosing a time point for analyzing and intervening, there is always a trade-off that must be faced: on the one hand, during early differentiation, cell lineages are more susceptible to fate manipulation but have fewer discernable image features; on the other hand, during later differentiation, cell fates are more fixed but their heterogeneity can be observed more easily. Therefore, it is essential to tailor the strategy design depending on the nature and the image characteristics of a specific PSC differentiation.

Several issues seem likely to be encountered while applying our strategy to other PSC differentiation processes. First, acquiring manually labeled data for training ML models is often a cumbersome process, so it is desirable to explore other ML paradigms, including self-supervised learning^82^ and few-shot learning^83^, which will probably enable models to learn the cell heterogeneity with fewer labeled data. Second, cell images are also susceptible to many factors (e.g., cell line, cell passage number, cell state) which inevitably result in a data distribution change, and may damage the predictive performance of ML models. Several approaches, such as sample weighting^84^ and risk extrapolation^85^, have been introduced to improve a model’s out-of-distribution generalization ability, which could be further explored. Our strategy possesses compatibility with other technologies (e.g., phototoxic dyes^86^ or the local light-responsive heat material^87^ in cell killing for in situ purification, automated cell culture machines^88,89^), which promisingly can be integrated into a self-adaptive and closed-loop system for full-automatic processing of PSC differentiation in vitro.

## Materials and methods

### Human PSCs culture

All pluripotent and differentiation cells were maintained at 37 °C in the cell incubator (LS-C0150, Thermo), with 5% CO_2_ and atmospheric (21%) O_2_. Human induced pluripotent stem cell (iPSC) lines iPS-18 (Y00300, TaKaRa), iPS-B1 (CA4025106, CELLapy), iPS-M (RC01001-A, Nuwacell), iPS-F (RC01001-B, Nuwacell), and human embryonic stem cell (ESC) lines WIBR3 (Whitehead Institute Center for Human Stem Cell Research, Cambridge, MA)^90^ and H9 were routinely cultured in PGM1 medium (CELLapy) or mTeSR1 medium (Stemcell Technologies) on growth factor-reduced Matrigel-coated (Corning) plate. iPSCs and ESCs were passaged every 3–5 days using Versene (Gibco) depending on the cell density. 3–5 μM Y27632 (Selleck Chemicals) dissolved in dimethyl sulfoxide (DMSO) was added for the first 12–24 h after passage. The pluripotent cells were seeded in 6-well plates.

### Cardiac differentiation of PSCs

PSCs were split into E8 medium (Cauliscell Inc) at the ratio of 1:10 or 1:12 for the last passage before beginning differentiation. Cells differentiated in 24-well plates or 96-well plates. When they reached ~80–90% confluence, the medium was changed to RPMI + B27– medium, consisting of RPMI 1640 (RPMI, Gibco), 2% B27 without insulin (Gibco), and 1% Penicillin-Streptomycin (Life Technologies)^20^. At stage I (from PSC to mesoderm, day 0–3), PSCs were initially treated with CHIR (Selleck Chemicals). CHIR concentration and duration depended on the cell lines and experiment design, ranging from 2–20 μM and 24–48 h, respectively. After CHIR treatment, the medium was replaced with RPMI + B27– medium until 72 h (day 3). At stage II (CPC stage, day 4–6), the RPMI + B27– medium was renewed and supplemented with 5 μM IWR1 (Selleck Chemicals) for 48 h. CPCs could be induced on day 6. At stage III (CM stage, day 7–~12), CPCs continued their differentiation process to CMs in RPMI + B27 medium, consisting of RPMI 1640 (RPMI, Gibco), 2% B27 with insulin (Gibco), and 1% Penicillin-Streptomycin (Life Technologies). The medium was replaced with fresh RPMI + B27 medium every 3 days. Beating cells could be noted from day 8–9. S12 medium (RPMI + S12) could also support efficient CM differentiation, see reference for S12 medium details^54^.

For manipulating the initial PSC clone size, we changed the enzyme digestion time, and forces and times of pipetting during passaging before differentiation, while the number of initial PSCs was strictly identical after passage for each well.

As for switching the CHIR concentration in the middle of CHIR treatment at stage I, the previous medium remained unchanged if possible (i.e., directly adding CHIR while switching to a high concentration and adding RPMI + B27– while switching to low concentration).

### Purification of PSC-CMs

On differentiation day 10–12, CMs were washed with DPBS (HyClone) to remove RPMI + B27 medium, and then changed to Glc and Gln-free DMEM (no Pyr, no Lac, and no fatty acids) (Gibco), supplemented with 4 mM L-lactic acid (Selleck Chemicals) for 3–6 days. The medium was renewed every 2–3 days to eliminate dead cells^91^. The metabolic selection method for PSC-CMs was used in the sample preparation for quantitative real-time PCR (RT-PCR).

### Immunofluorescence staining

Cells were washed with PBS and fixed in 4% paraformaldehyde (DING GUO) for 15 min at room temperature, permeabilized with 0.1% Triton X-100 (Amresco) for 15 min at room temperature, blocked in 3% Normal Donkey Serum (Jackson) and 0.1% Triton X-100 for 30 min at room temperature, and incubated with primary antibodies overnight at 4 °C in PBS plus 0.1% Triton X-100 and 3% Normal Donkey Serum (Jackson). Cells were washed three times with PBS on the next day and then incubated with secondary antibodies in PBS with 1% bovine serum albumin (BSA) for 1 h at 37 °C in a dark environment. Cells were washed again as above. Nuclei were stained with Hoechst 33342 (Yeasen). An Axio vert A1 Microscope (Zeiss) or Celldiscoverer 7 (Zeiss) was used for imaging. Details of antibody are provided in Supplementary Table S5.

### Electrophysiological recording

Action potentials and calcium currents were recorded by a Whole-cell patch clamp using an Axon 700B patch-clamp amplifier (Axon Instruments, USA). Action potentials were recorded in current-clamp mode. Pipette resistances were 2–3 MΩ. The external solution contained (in mmol/L): 140 NaCl, 5.4 KCl, 1.8 CaCl_2_, 1 MgCl_2_, 10 glucose, and 10 Hepes (pH 7.4 adjusted with NaOH). The pipette solution contained 120 KCl, l MgCl_2_, 3 MgATP, 10 Hepes, and 10 EGTA (pH 7.2 adjusted with KOH).

Calcium currents were recorded in voltage-clamp mode. Pipette resistances were 2–3 MΩ. The external solution contained (in mmol/L): 135 NaCl, 5 CsCl, 1.8 CaCl_2_, 1 MgCl_2_, 1.2 NaH_2_PO_4_, 10 glucose, 10 Hepes, and 20 μM TTX (pH 7.4 adjusted with NaOH). The pipette solution contained 120 CsCl, l MgCl_2_, 5 MgATP, 20 TEACl, 10 Hepes, and 5 EGTA (pH 7.2 adjusted with CsOH).

### Calcium imaging recording

PSC-CMs were incubated with 5 μM fluo-4 AM for 10 min, then washed three times with external solution, and imaged with Zeiss LSM-510 confocal microscope equipped with 40× oil lens. The external solution contained (in mmol/L): 140 NaCl, 5.4 KCl, 1.8 CaCl_2_, 1 MgCl_2_, 10 glucose, and 10 Hepes (pH 7.4 adjusted with NaOH). The fluo-4 fluorescence was excited by a 488 nm laser, filtered by a 505 nm long-pass filter, and recorded in line-scan mode at 3.84 ms/line.

### Region-selective cell purification based on a photoactivatable probe

DACT-1 was dissolved in DMSO to a concentration of 10 mM and aliquoted to store at –20 °C. Cells were incubated with RPMI + B27 medium containing 1 μM DACT-1 for 30 min at 37 °C. Photoactivation experiments were performed on an inverted fluorescence microscope (Nikon-TiE) equipped with a motorized stage (Marzhauser SCAN IM). Imaging was performed with a 20 × 0.75 NA dry objective, a spinning disk confocal unit (Yokogawa CSU-X1), and a scientific Complementary Metal Oxide Semiconductor (CMOS) camera (Hamamatsu ORCA-Flash 4.0 v2). For DACT-1-based region-selective photoactivation, DIC images of cells in 96-well plates were inspected and then the snapshots of cells were taken for selection. The region of interest (ROI) was selected and drawn as polygons in MATLAB (R2018b, MathWorks). Parallel horizontal trace lines with 20-μm spacing were generated to intersect the polygons, and the stage coordinates of the intersection points were calculated. A 405-nm laser line (Coherent OBIS 405 nm, 50 mW) was focused on the sample plane with the objective to form a spot 20 μm in diameter. The output power of the laser line was set to 10% (i.e., 5 mW) to reduce photodamage. Then the motorized stage with the light spot was set to move according to trace lines at 0.12 mm/s in order that the 405-nm focal illumination could scan across the ROI to photoactivate cells restrictedly. Photoactivation of each ROI was typically completed within 1 min. The microscope, camera, and lasers were controlled with Micro-Manager (version 2.0.0). For customized hardware control (e.g., stage movement), MATLAB was used to run the Micro-Manager GUI and core. After irradiation, DACT-1-labeled cells can be detected by confocal imaging using a 561-nm laser line (Coherent OBIS 561 nm, 50 mW).

After selective photoactivation, cells were dissociated using 0.05% Trypsin-EDTA (Gibco) at 37 °C for 5–7 min, which depended on the days of differentiation, followed by gentle shaking in a 37 °C incubator for 2 min. After dispersion, the cells were filtered through a 40-μm cell strainer (Biologix). Then the cells were centrifuged at 850 rpm for 3 min and resuspended in 0.5% BSA. The DACT-1^+^ and DACT^–^ cells were separated and collected from BD FACSAria III Cell Sorter (Becton Dickinson). The sorted cells were resuspended in RPMI + B27 medium with 10% FBS (Vistech) and 5 μM Y27632 and transferred onto Matrigel-coated plates. The medium was changed to RPMI + B27 medium on the second day. Cells were cultured in RPMI + B27 medium for 6 days and then were fixed for cTnT immunostaining.

### Hoechst/PI staining

For the Hoechst/PI staining, cells were treated with RPMI 1640 + B27– supplemented with 12 μM CHIR with or without BI-1347 for 48 h, then were incubated with RPMI 1640 + B27– supplemented with 5 μg/mL Hoechst 33342 (Yeasen), maintained at 37 °C for 5 min. 100× PI stain Solution (Coolaber, SL7091) was diluted with PBS and cells were incubated with diluted PI for 10 min at room temperature. Cells were immediately imaged using Celldiscoverer 7.

### RT-PCR

Total RNA was isolated using the EasyPure RNA Kit (TransGen), and cDNA was synthesized using the TransScript All-in-One First-Strand cDNA Synthesis SuperMix for the qPCR kit (TransGen). Quantitative real-time PCR was performed using ChamQ SYBR qPCR Master Mix (Vazyme). The gene expression levels were normalized using GAPDH as an internal reference. Primer sequences are listed in Supplementary Table S6.

### RNA-seq

A total of 12 samples of IR-CPC, non-CPC, CM, and iPSC (including three biological repetitions) were collected and analyzed together, in which IR-CPCs were purified through DACT-1 photoactivation method; non-CPC were the cells under inappropriate CHIR conditions on day 6. iPS-B1 cell line was used in sample collection. Data were shown in Supplementary Fig. S9a–f.

A total of 10 samples including an iPSC sample and cells under different CHIR doses (CHIR 2 μM 48 h, 6 μM 24 h, 6 μM 36 h, 10 μM 24 h, 8 μM 36 h, 6 μM 48 h, 12 μM 24 h, 12 μM 36 h, and 10 μM 48 h) were collected before withdrawing CHIR. iPS-18 cell line was used in sample collection. Data were shown in Supplementary Fig. S11a–d.

A total of nine samples of iPSC-CM with BI-1347, iPSC-CM without BI-1347, and iPSC (including three biological repetitions) were collected and analyzed together. iPSC-CM with BI-1347 (induced by CHIR 12 μM 48 h and BI-1347 0.5 μM 48 h) and iPSC-CM without BI-1347 (induced by CHIR 8 μM 48 h) were collected after 12 days of cardiac differentiation. iPS-B1 cell line was used in sample collection. Data were shown in Fig. 6i and Supplementary Fig. S13a, b.

A total of 14 samples were collected and analyzed for exploring the mechanism of BI-1347 (including two biological repetitions), including two iPSC samples and 6 sample pairs from 2 groups, in which CM was induced in the presence or absence of BI-1347 (0.5 μM, 48 h) under the overdose CHIR (16 μM, 48 h). Guided by this experimental design, cells were collected at three time points (24 h, 48 h, and 72 h). iPS-B1 cell line was used in sample collection. Data were shown in Supplementary Fig. S14e–g.

RNA was extracted using the EasyPure® RNA Kit (TransGen), then sequenced on NovaSeq6000-PE150. The reads were processed and mapped to the Homo Sapiens genome GRCh38/hg38. The figures of PCA, heatmaps, and GO enrichment were visualized by Omicshare Tools (https://www.omicshare.com/). Heatmaps represent log_2_(FPKM + 1) normalized over samples. The detection of the differentially expressed genes (DEG calling) between samples was performed with DESeq^92^ when samples included multiple repetitions. Only *P* value < 0.05, fold change < 0.5 or > 2, and mean expression > 1 were regarded as DEGs and used in further analysis. DEG calling was performed with expression fold change in samples without repetition. Only fold change < 0.5 or > 2, and mean expression > 1 were regarded as DEGs. The front-rank DEGs were enriched for GO analysis.

More details can be accessed through GEO accession: GSE226159.

### Image acquisition

The live-cell bright-field images within the whole CM differentiation process were acquired using Celldiscoverer 7 (an imaging system equipped with an inverted research microscope for long-term cell culture observations, made by Carl Zeiss). The ORCA-Flash 4.0 V3 digital CMOS camera with 2048 × 2048 pixels could achieve automatic live-cell imaging. During differentiation, the petri dish was maintained in an internal incubator inside the microscope at 37 °C with 5% CO_2_.

The 96-well, 24-well, and 384-well plates (Falcon) were used. After autofocus, bright-field images were taken under a 5× objective and a 2× tube lens. A 2 × 2 binning was applied to each acquisition to improve the signal-to-noise ratio and reduce the storage space, thus generating 1024 × 1024-pixel images. The scaling resolution was 1.3 μm per pixel according to the previous light path design.

Each well in the 96-well plate was imaged with 25 tiles in a 5 × 5 pattern with an overlap of 5%–15% on adjacent fields. After stitching, the tiles of each well generated an image with 4860 × 4860 pixels, 6.3 mm × 6.3 mm in size. To ensure the sharpness of the images, 3–5 Z-stacks of images with equidistant intervals (3–6 μm) were used for further in-focus image selection. For three typical Z-stacks of images, a whole 96-well plate scanning takes ~1.2 h for 7200 (5 × 5 × 3 × 96) tiles in one round. For the following analysis, only images of 9 tiles in a 3 × 3 pattern located in the center of the well were used as “whole-well images” to avoid the interference of images containing well edges.

For the 24-well plate, similarly, 156 tiles were stitched into a 20,284 × 20,284-pixel rectangle image for each well. In particular, for some wells at the edge of the plate, only 136 tiles were taken because of the limitation of the shooting range of the objective lens. An approximate 13.0 mm × 13.0 mm visual range was obtained for each well. A total of 10,992 (136 × 3 × 4 + 156 × 3 × 20) images were collected in one round.

For the 384-well plate (square well), 9 tiles in a 3 × 3 pattern with an overlap of 10% were taken. The scaling resolution was 1.3 μm per pixel with the same objective and camera. In total, 3456 (3 × 3 × 1 × 384) images were collected for image acquisition in one round.

The cell images were automatically saved in CZI format generated by ZEN software (Zeiss company supporting picture acquisition software, version V2.0-V3.1 was used). Images were automatically exported to uncompressed TIFF format or PNG format for real-time image processing.

### Visualization of the local image features of the overall differentiation process

The local image features of the overall CM differentiation process were first analyzed by PCA and LDA^42^. The dataset consisted of *n* = 19,968 images (for 96 wells at 208 time points). The feature vector for each image was obtained from SIFT^39^, SURF^40^, and ORB^41^ feature descriptors. SIFT, SURF, and ORB was first used to extract keypoints from the image and compute a feature vector (of 128-D, 64-D, and 32-D, respectively) for each keypoint; then for every image, the mean and standard deviation of each dimension of all the keypoints’ feature vector were computed, resulting in a (128 + 64 + 32) × 2 = 448-D vector. PCA was applied to project the feature vectors to the 2-D plane spanned by the first two principal components (denoted by PC1 and PC2). LDA was applied to find a 2-D plane (spanned by the first two linear discriminants, LD1 and LD2) which can best separate wells with an underdose, optimal dose, and overdose of CHIR. Extraction of local features, PCA, and LDA were implemented using the OpenCV^93^ and the scikit-learn package^94^ in Python.

### Evaluation of differentiation efficiency using cTnT fluorescent labels

The PSC-to-CM differentiation efficiency of a well was quantified by the average intensities of the final fluorescence images. Concretely, for a *W* × *W* fluorescence image *I* (with intensity values in [0, 1]), its “Differentiation Efficiency Index” was defined by the total intensity values greater than a threshold *α*, i.e.,

$${{{\mathrm{Differentiation}}}}\,{{{\mathrm{Efficiency}}}}\,{{{\mathrm{Index}}}} = \frac{1}{{W^2}}\mathop {\sum}\limits_{\begin{array}{*{20}{c}} {1 \le i,j \le W,} \\ {I_{i,j} > \alpha } \end{array}} {I_{i,j}} ,$$

where 1/*W*^2^ is a normalizing factor. In all of our experiments, *α* was chosen to be 0.5.

### Deep learning for cTnT fluorescence prediction on bright-field images at CM stage

The pix2pix model^43^ was utilized to predict cTnT fluorescent labels from bright-field images. Pix2pix is a classic model for supervised image-to-image translation based on conditional adversarial networks. In the pix2pix model, the generator *G* learns to predict the fluorescence labels from the bright-field images, and the discriminator *D* learns to distinguish between fake and real “bright-field-fluorescence” image pairs (Supplementary Fig. S3a). Formally, let *x* and *y* be the bright-field image and the corresponding fluorescence image, respectively, and let *z* represent the randomness in generator *G*. Then the final training objective is a *L*_1_ reconstruction loss (weighted by *λ*) plus an adversarial term:

$$G^ \ast = \arg \mathop {{\min }}\limits_G \mathop {{\max }}\limits_D \lambda {{{\mathcal{L}}}}_{L_1}\left( G \right) + {{{\mathcal{L}}}}_{{{{\mathrm{cGAN}}}}}\left( {G,D} \right),$$

where

$${{{\mathcal{L}}}}_{L_1}\left( G \right) = {\Bbb E}_{x,y,z}\left[ {\left\| {y - G\left( {x,z} \right)} \right\|_1} \right],$$

$${{{\mathcal{L}}}}_{{{{\mathrm{cGAN}}}}}\left( {G,D} \right) = {\Bbb E}_{x,y}\left[ {\log D\left( {x,y} \right)} \right] + {\Bbb E}_{x,z}\left[ {\log \left( {1 - D\left( {x,G\left( {x,z} \right)} \right)} \right)} \right].$$

Following the original design^43^, the generator *G* is based on the U-Net architecture^95^ (Supplementary Fig. S3b). The transposed convolution operation was replaced with nearest neighbor up-sampling followed by convolution operation to avoid checkerboard artifacts^96^. Instance normalization was used in the generator. The discriminator *D* is a patch discriminator where each pixel in the output score map has a receptive field of size 16 × 16 pixels (Supplementary Fig. S3c).

The dataset configuration for training and testing was listed (Supplementary Table S1). All the images were resized to 1536 × 1536 pixels. During training, in each epoch, a total of 1260 patches (of size 256 × 256 pixels) were randomly selected from the bright-field and fluorescence image pairs in the training set and passed to the network with a mini-batch size of 16. The pix2pix model was trained for 2000 epochs with the Adam optimizer^97^ with *β*_1_ = 0.5, *β*_2_ = 0.999. *λ* was chosen to be 100. The learning rate was kept as 0.0002 in the first 1000 epochs and linear decayed to 0 in the last 1000 epochs. The *L*_1_ loss, $\lambda {{{\mathcal{L}}}}_{L_1}\left( G \right)$ (scaled by 1/5) and the GAN loss, log (1 − *D*(*x*, *G*(*x*, *z*))) of the generator during training were shown (Supplementary Fig. S4a). To ensure the fidelity of fluorescence prediction, GAN loss was disabled in the last 1000 epochs of training.

During testing, whole-well bright-field images were directly passed to the generator network. We evaluated the performance by comparing the predicted and the ground-truth fluorescence intensity at the pixel level, providing a heatmap and a Pearson correlation value *r* (Fig. 2d); here fluorescence images were resized to 512 × 512 pixels, and the numbers in the bins of the heatmap are frequency counts per 100. We also computed the image-level correlation between the true and predicted fluorescence in terms of the Differentiation Efficiency Index (Fig. 2e, f). The training and testing of the pix2pix model were implemented using the PyTorch framework^98^ and trained on a PC with an 11 GB NVIDIA RTX 2080Ti GPU.

### Weakly supervised learning for CPC recognition at the CPC stage

The CPC regions in day 6 bright-field images were manually annotated by tracking the location of cTnT^+^ cells in the image streams (from day 12 back to day 6) and the expert experience about CPC image textures, represented by a CPC segmentation mask (Supplementary Fig. S5a). The CPC mask contained cell regions labeled with dark gray (cells successfully differentiate into CMs), light gray (cells likely differentiate into CMs), and black (cells fail to differentiate into CMs).

The recognition of CPC was based on weakly supervised learning. In the training stage (Supplementary Fig. S5a), the ResNeSt-101^49^ network was trained to classify the bright-field image patches labeled with “positive” or “negative”. Day 6 bright-field images (resized to 2816 × 2816 pixels) were cropped to local patches (512 × 512 pixels) with a 50% overlap between adjacent patches. These patches were labeled with positive (≥ 30% dark gray regions in the mask) or negative (only black regions in the mask); patches in other cases were discarded. Finally, 8463 labeled patches (3175 positive and 5288 negative) were extracted from the bright-field images. 20% of the training image patches were used for validation. The ResNeSt-101 network was trained for 300 epochs, using the Adam optimizer^97^ with a mini-batch size of 6 and a learning rate of 0.00003. The change of training loss and validation loss, with ACC (accuracy) and AUC (the area under the curve) on the training set, were also shown (Supplementary Fig. S6a, b).

In the testing stage (Supplementary Fig. S5b), for new bright-field images of day 6, we used the trained ResNeSt-101 with the Grad-CAM^50^ approach to generate the prediction of CPC regions. In the test set, the day 6 bright-field images were cropped to patches (of size 512 × 512 pixels) with a 75% overlap between adjacent patches. The trained network then made predictions on these patches. For a patch predicted as positive, its heatmap was computed from the class-activation maps weighted by gradients. Concretely, let *A*^*k*^ (where *k* is the channel index) be the feature map activations of the convolutional layer right before the global average pooling operation in the ResNeSt-101. The importance weight *α*_*k*_ was derived from the gradient of the score for class “positive” (before soft-max, denoted by *y*_+_), with respect to the feature map *A*^*k*^:

$$\alpha _k = \frac{1}{Z}\mathop {\sum}\limits_i {\mathop {\sum}\limits_j {\frac{{\partial y_ + }}{{\partial A_{ij}^k}}} } ,$$

where *Z* is the number of pixels of the feature map. The heatmap was then defined by ${{{\mathrm{ReLU}}}}\left( {\mathop {\sum}\nolimits_k {\alpha _kA^k} } \right)$. For a patch predicted as negative, its heatmap was simply set to all-zero. These heatmaps were normalized to 0–255 and binarized with a threshold of 10 to yield a binary prediction of CPC regions. The resulting patch-level predictions (heatmaps and predicted CPC regions) were finally reconstructed to whole-well (2816 × 2816 pixels) predictions. The dataset configuration for training and testing were listed (Supplementary Table S2).

The performance of weakly supervised localization of CM-committed CPCs was evaluated by a pixel-level comparison between the predicted CPC masks and the annotated CPC masks (Fig. 3e), as well as an image-level comparison between the predicted percentage of CPC regions and the final Differentiation Efficiency Index (computed from the day 12 cTnT fluorescent label) (Fig. 3f, g). For the pixel-level comparison, dark gray and light gray regions in the CPC mask were regarded as the ground-truth CPC regions and recolored with white. We counted the “true negative”, “true positive”, “false negative”, and “false positive” pixels in the binary prediction of CPC mask, denoted by “TN”, “TP”, “FN”, and “FP”, respectively. Accuracy, F1 score, precision, recall, specificity, and intersection-over-union (IoU), were defined by

$${{{\mathrm{accuracy}}}} = \frac{{{{{\mathrm{TN}}}} + {{{\mathrm{TP}}}}}}{{{{{\mathrm{TN}}}} + {{{\mathrm{FN}}}} + {{{\mathrm{TP}}}} + {{{\mathrm{FP}}}}}},$$

$${{{\mathrm{precision}}}} = \frac{{{{{\mathrm{TP}}}}}}{{{{{\mathrm{TP}}}} + {{{\mathrm{FP}}}}}},$$

$${{{\mathrm{recall}}}} = \frac{{{{{\mathrm{TP}}}}}}{{{{{\mathrm{TP}}}} + {{{\mathrm{FN}}}}}},$$

$${{{\mathrm{F}}}}1\,{{{\mathrm{score}}}} = \frac{{{{{\mathrm{TP}}}}}}{{{{{\mathrm{TP}}}} + ({{{\mathrm{FN}}}} + {{{\mathrm{FP}}}})/2}},$$

$${{{\mathrm{specificity}}}} = \frac{{{{{\mathrm{TN}}}}}}{{{{{\mathrm{TN}}}} + {{{\mathrm{FP}}}}}},$$

$${{{\mathrm{IoU}}}} = \frac{{{{{\mathrm{TP}}}}}}{{{{{\mathrm{TP}}}} + {{{\mathrm{FP}}}} + {{{\mathrm{FN}}}}}}.$$

Images fully covered by CPCs or without CPCs were discarded to avoid meaningless values. For the image-level comparison, Pearson correlation analysis was conducted between the predicted percentage of CPC regions and the true Differentiation Efficiency Index.

Manual annotation of the CPC masks, cropping of bright-field images, and reconstruction of the whole-well prediction were implemented in MATLAB (R2018b, MathWorks). ResNeSt-101 and Grad-CAM were implemented using the PyTorch framework^98^ and run on a PC with an 8 GB NVIDIA Quadro P4000 GPU.

### ML for CHIR dose assessment using 0–12 h bright-field image streams

CHIR concentration and duration of high-efficiency wells (cTnT^+^ cell percentage ≥ 50%) were negatively correlated within one batch, with Spearman’s rank correlation *ρ* = –0.80 ± 0.14 (*n* = 8 batches); titration results of three typical batches were shown (Fig. 4a).

The dataset for CHIR dose assessment contained 0–12 h whole-well bright-field image streams (Supplementary Table S3). The image streams consisted of 10 bright-field images (denoted by time points T1, T2, …, T10) uniformly taken during 0–12 h. The images were resized to 4860 × 4860 pixels. For each CHIR duration (24 h, 36 h, or 48 h), CHIR concentrations with a mean percentage of cTnT^+^ cells ≥ 20% were identified as optimal concentrations range [*c*_1_, *c*_2_]. CHIR concentrations *c* outside [*c*_1_, *c*_2_] were labeled as “low” (if *c* < *c*_1_) or “high” (if *c* > *c*_2_). The “ΔCHIR Concentration” of each concentration level *c* was defined by its relative difference to [*c*_1_, *c*_2_], i.e.,

$${\Delta}{{{\mathrm{CHIR}}}}\,{{{\mathrm{Concentration}}}}\left( c \right) = \left\{ {\begin{array}{*{20}{l}} {c - c_1,} \hfill & {c < c_1} \hfill \\ {0,} \hfill & {c_1 \le c \le c_2} \hfill \\ {c - c_2,} \hfill & {c > c_2} \hfill \end{array}} \right.,$$

which can measure the degree of deviation from the optimal conditions.

These bright-field image streams were then represented by 21-D handcrafted feature vectors. Since SIFT, SURF, ORB feature descriptors could not distinguish the bright-field images in the low-efficiency and high-efficiency groups at stage I (Supplementary Fig. S2b), we used other features including Fractal Dimension, cell colony statistics (Area, Circumference, Area-Circumference Ratio, Brightness, Local Entropy), and Optical Flow. Among these features, Optical Flow (referred to as Type-II feature) was computed for every two consecutive time stamps, while the others (referred to as Type-I features) were computed for every time stamp. The feature sequences of Area, Circumference, Area-Circumference Ratio (A-C Ratio), and Optical Flow were normalized by dividing the values of the first value in the sequence. T1–T10 were divided into pre-phase, mid-phase, and post-phase, and features in the same phase were averaged out. Therefore, three numbers are computed for each of the 7 features, resulting in a 21-dimensional feature representation (Supplementary Fig. S10a). The computational details of each feature are listed below.

1. Fractal Dimension. The Fractal Dimension measures the roughness and self-similarity of an image. The differential box-counting approach^99^ was used to obtain the fractal dimension (ranging from 2–3), with box widths chosen to be $2,2k,2k^2, \ldots ,2k^{15}$ where $k = \left( {243} \right)^{1/15}$.
2. Local Entropy. For each pixel, we computed the entropy of the intensity distribution of neighboring pixels (with a Euclidean distance ≤ 10 pixels). Pixels with a local entropy ≥ 3 were identified as covered by cells. The Local Entropy of an image was defined by the averaged local entropy of the cell-containing regions.
3. Area, Circumference, and A-C Ratio. They were derived from the cell-containing regions, and identified using the local-entropy criteria. The A-C Ratio can reflect the fraction of cells located at the periphery of colonies.
4. Cell Brightness. Cell Brightness is the average intensity value of cell-containing regions, which may be related to the compactness of the cells.
5. Optical Flow. Optical flow measures the cell movements during differentiation, which reflects the contraction velocities of cell colonies. Gunner Farneback’s algorithm^55^ was used to estimate the dense optical flow field for images at two consecutive time points with a window size of 16. Flow vectors with a magnitude ≤ 4 are discarded. The average magnitude of the optical flow vectors was defined as the Optical Flow of an image.

The high-dimensional feature vectors (21-D if using all the features, or 4-D if using the selected features only) were visualized by dimensionality reduction techniques: LDA (Fig. 4d; Supplementary Fig. S10e) and PCA (Supplementary Fig. S10c, d)^42^. LDA was applied to verify the discriminative ability of the feature representation, and PCA for visualizing the sample distribution. The shrinkage parameter for LDA was set to 0.1 and 0 when visualizing the 21-D and 4-D feature spaces, respectively.

The logistic regression model was utilized for the classification task. The training data were reweighted to handle the class imbalance problem. When using all the 21 features, *l*_1_ regularization with coefficients of 1/4, 1/8, and 1/8 (for CHIR duration = 24 h, 36 h, and 48 h, respectively) was used to encourage sparse parameters; when using only 4 selected features, the *l*_2_ regularization with a coefficient of 0.1 was used. The final loss functions were optimized using the liblinear solver. Accuracy, precision, recall, F1 score, and AUC were used to evaluate the performance of logistic regression. Precision, recall, F1 score, and AUC were macro-averaged over the three categories.

The logistic regression model could also provide a “Deviation Score” for each CHIR concentration level *c* by averaging the predictions of wells with concentration *c* (Fig. 4f). Let *N*_*c*_ be the number of wells with concentration *c*, among which $N_c^{{{{\mathrm{low}}}}},N_c^{{{{\mathrm{optimal}}}}},N_c^{{{{\mathrm{high}}}}}$ wells are predicted as low, optimal, and high by logistic regression. Then, the Deviation Score is defined by

$${{{\mathrm{Deviation}}}}\,{{{\mathrm{Score}}}}\left( c \right) = \left( {N_c^{{{{\mathrm{high}}}}} - N_c^{{{{\mathrm{low}}}}}} \right)/N.$$

The Deviation Scores range from −1 to 1, which could reflect the deviation of the CHIR concentration from the optimal conditions. The predicted Deviation Scores computed from the test set of CD01-1 were compared with the true ΔCHIR concentrations for *n* = 20 CHIR doses (Fig. 4f).

To test the model’s generalization ability to new batches, a cross-batch validation was conducted under a CHIR duration of 24 h (Fig. 4g; Supplementary Fig. S10g, h). Feature selection was conducted to increase the generalization ability of the classification. In each train-test round, one batch was left for testing while others were for feature selection and training. The logistic regression model in each round was regularized by elastic-net (with *l*_1_-ratio chosen as 0.1 and weighted by 0.05) and optimized by the SAGA solver. The cross-batch validation was evaluated by the Pearson correlation value between the predicted Deviation Scores and true ΔCHIR Concentrations. Feature extraction, LDA, PCA, feature selection, and logistic regression were implemented by the Python-based package scikit-image^100^, scikit-learn^94^, and OpenCV^93^.

### ML for control of the initial PSC colony state

We prepared a dataset of *n* = 1934 whole-well bright-field images of the initial PSC colonies at 0 h (before CHIR treatment) (Supplementary Table S4). 343 features were extracted from the bright-field images to quantify the morphological profiles of the initial PSC colonies. The features were detailed as below.

1. Local Entropy, Cell Brightness, Cell Contrast, Total Variation. Local Entropy is the averaged local entropy of each pixel located in the cell-containing regions, where the local entropy of a pixel is computed from the intensity distribution of its neighborhood with radius = 5 pixels. Cell Brightness and Cell Contrast is the mean and the standard deviation of the intensities in the cell-containing regions. Total Variation is the *L*_1_ norm of the gradient of the bright-field images.
2. Hu Moment 1–7. They are the seven image moments^101^ of the bright-field images which were invariant to translation, scaling, and orthogonal transformations.
3. SIFT 1–256. They are a 256-D bag-of-keypoints representation^102^ using SIFT feature descriptor. Concretely, K-Means was first applied to obtain 256 clusters over all the keypoints’ SIFT feature vectors in 385 bright-field images (which were excluded from the dataset); then for each image in the dataset, we counted the number of keypoints assigned to each cluster, yielding a 256-D feature vector.
4. ORB 1–64. Similar to SIFT 1–256, ORB 1–64 are a 64-D bag-of-keypoints representation using ORB feature descriptor.
5. Area, Circumference, Area/Circumference Ratio. They are the total area, total circumference, and their ratio of the cell-containing regions. The area and the circumference are normalized, respectively, by width × width and width of the whole-well image.
6. Solidity, Convexity, Circularity. For a connected component *R*, solidity is defined by ${\textstyle{{{{{\mathrm{Area}}}}\left( R \right)} \over {{{{\mathrm{Area}}}}\left( {{{{\mathrm{convex}}}}\,{{{\mathrm{hull}}}}\,{{{\mathrm{of}}}}\,R} \right)}}}$; convexity is defined by ${\textstyle{{{{{\mathrm{Circumference}}}}\left( {{{{\mathrm{convex}}}}\,{{{\mathrm{hull}}}}\,{{{\mathrm{of}}}}\,R} \right)} \over {{{{\mathrm{Circumference}}}}\left( R \right)}}}$; circularity is defined by $4\pi {\textstyle{{{{{\mathrm{Area}}}}\left( R \right)} \over {\left[ {{{{\mathrm{Circumference}}}}\left( R \right)} \right]^2}}}$. For a bright-field image, its Solidity, Convexity, Circularity are, respectively, the average of the solidity, convexity, and circularity of all the connected components of its cell-containing regions, weighted by the connected component’s area.
7. Max Centroid-Contour Distance (CCD), Min CCD, Min/Max Ratio of CCD, Mean of CCD, Standard Deviation of CCD. For a connected component, the distribution of the distances between its centroid and boundary points are computed. Statistics (minimum, maximum, minimum/maximum ratio, mean, and standard deviation) are computed from the distribution. For a bright-field images, these features are also weighted averaged over all of the connected components of its cell-containing regions.
8. Spacing. To measure the spacing between cell-containing regions, cell-free regions were skeletonized and the average distance between the skeletons and the cell-containing regions were computed as the spacing.

To analyze how the morphological profiles of the PSC colonies contribute to the final differentiation efficiency under optimal CHIR conditions, the day 12 cTnT fluorescence images were captured and used to determine the optimal CHIR conditions for each batch. Since differentiation potential varies among different cell lines even under optimal CHIR conditions, the Differentiation Efficiency Index for each well was normalized by the maximum Differentiation Efficiency Index in its cell line.

We built a random forest regression model to predict the final Differentiation Efficiency Index from the 343 features of the initial PSC colonies. 1350 wells were used for training and 584 wells were for testing. To determine the feature importance (Fig. 5d), 1000 decision trees with a maximum depth of 8 were used in the random forest model. 15 features were considered at each split in the decision tree. For efficiency prediction (Fig. 5g), 20 decision trees were used in the random forest model. Quantification of the PSC colony states and the random forest regression model were implemented by the Python-based package scikit-image^100^, scikit-learn^94^, and OpenCV^93^.

### Application of the image-based ML for small molecule screening

Cells from iPS-B1 line were seeded on 384-well plates and induced to CM following the previous protocol^20^. Regarding the preparation of 0.2 mM small-molecule libraries, the chemical libraries arrayed in 96-well plates consisted of a Kinase Inhibitor Library with 203 compounds (MedChem Express), an Epigenetics Inhibitor Library with 135 compounds (Thermo), an Approved Drug Library with 1700 compounds (Thermo), a Clinical Compound Library with 250 compounds (Thermo), a Metabolism Inhibitor Library with 132 compounds (MedChem Express), and an in-house library with ~600 compounds (purchased from MedChem Express or Selleck Chemicals). All the compounds were transferred in a volume of 0.3 μL to 384-well plates, making a final working concentration of 2 μM. All the compounds were added with CHIR (16 μM) for 0–48 h.

Both negative control (CHIR 16 μM with DMSO) and positive control (CHIR 6 μM with DMSO) were also included in the 384-well plates. We collected the bright-field images on day 6 and used the trained ResNeSt model with Grad-CAM to predict the percentage of CPC regions for wells added with different small molecules. The bright-field images were cropped from the center part of whole-well images and resized to 1792 × 1792 pixels in accordance with the scale during the model training. These images were divided into patches (of size 512 × 512 pixels) with a 50% overlap between adjacent patches. After predicting, small molecules were ranked by predicted percentage of CPC regions and the top 40 compounds were regarded as initial candidates. These candidates were further confirmed by assessing their effects on broadening the effective CHIR doses in 96-well plates.

### NPC differentiation and early assessment of CHIR doses using day 4 bright-field images

The differentiation of NPCs followed a published protocol^19^. PSC and ESC were resuspended in PGM1 medium (CELLAPY), and seeded in a 24-well Matrigel-coated (Corning) plate with 10 μM Y27632 (Selleck Chemicals). From day 0, the medium was changed to Advanced RPMI-1640 (Gibco) with 1% Penicillin-Streptomycin (Life Technologies) and 1% GlutaMAX supplement (Gibco). The medium was added with 2–15 μM CHIR (Selleck Chemicals) for 4 days (day 0–4), followed by 3 days treatment of 10 ng/mL Activin A (day 5–7), and then 2 days of 10 ng/mL FGF9 (day 8–9). On day 9, cells were collected for immunofluorescent staining of SIX2.

We prepared a dataset of day 4 bright-field images of nephron progenitor cells, labeled with “low”, “optimal”, and “high” identified from the CHIR dose condition and the final immunofluorescent results. The dataset was randomly divided into a training set (*n* = 3398) and a test set (*n* = 1457). We extracted local features for the bright-field images using a 256-D “bag-of-keypoints” feature vector derived from the SIFT feature descriptor^102^. T-SNE was used to visualize the features, with perplexity chosen to be 60 (Supplementary Fig. S15d).

Logistic regression was used to classify the bright-field images in the “low”, “optimal”, and “high” CHIR dose groups based on the local features. The training data were reweighted to handle the class imbalance problem. The logistic regression model was trained with *l*_1_-regularization weighted by 1, and optimized by liblinear solver. Accuracy, precision, recall, F1 score, and area-under-the-curve (AUC) were used to evaluate the performance of logistic regression. Precision, recall, F1 score, and AUC were macro-averaged over the three categories (Supplementary Fig. S15e).

### Hepatic Differentiation and recognition of DE

The differentiation of DE referred to an established protocol for hepatocyte-like cell induction based on small molecule compound^103^. Briefly, iPS-B1, iPS-18, and iPS-M were seeded in a 24-well plate and cultured in PGM1 medium. While PSC reached the desired confluence, the medium was changed to RPMI + B27– medium supplemented with CHIR and IDE1 (MedChem Express). After 24 h, the medium was changed to RPMI + B27– medium containing IDE1 of the previous concentration for 2 days. In order to get results with different efficiencies, PSC confluence, CHIR concentration, and IDE1 concentration were fine-tuned according to the experiment design in a few wells. The medium was changed every day. On day 3 (DE stage), cells were fixed for immunofluorescent staining of SOX17. Live-cell bright-field images and SOX17 fluorescence images were captured.

We applied the weakly supervised learning model for the recognition of DE cell regions. Since SOX17 localized in the cell nucleus, the SOX17^+^ cell regions were obtained by morphological closing of the binarized SOX17 fluorescence images. The training dataset included 8 whole-well bright-field images (resized to 16,000 × 16,000 pixels), which were cropped to patches (512 × 512 pixels), with an overlap of 25% between adjacent patches. According to the SOX17 fluorescence results, these patches were labeled as “positive” (≥ 20% SOX17^+^ cell regions), “negative” (without SOX17^+^ cell regions), or otherwise excluded from the training set.

After trained for 300 epochs on these labeled patches, the model was tested on 45 new bright-field images (of size 5120 × 5120 pixels), which were cropped to patches (512 × 512 pixels) with an overlap of 50% between adjacent patches. Grad-CAM heatmaps for each bright-field images were reconstructed from patch-level results.

### Statistical analyses

Data are expressed as means ± SD. *P* values < 0.05 were considered significant for all statistical tests. Statistical analyses of differences between experimental groups were performed with GraphPad Prism 8/9, using two-tailed Student’s *t*-tests or one-way ANOVA (followed by Dunnett’s multiple comparisons test). The two-tailed Pearson or Spearman correlation analysis was performed by Prism 8/9 and Python’s scipy package^104^. The *P* values of the one-way ANOVA F-test in the feature selection were computed by Python’s scikit-learn package^94^. All of the statistical tests performed are indicated in the figure legends.

## Supplementary information

Supplementary information

Supplementary Video S1

Supplementary Video S2

Supplementary Video S3

Supplementary Video S4

Supplementary Video S5

## Data Availability

Bulk RNA-seq data generated in this study have been deposited in NCBI GEO with the accession code GSE226159. Single-cell RNA-seq data of human fetal heart development used for Supplementary Fig. S9f are available in the NCBI GEO repository: Cui et al.^53^ GSE106118. The image datasets used for training and testing the ML models are available in the GitHub repository https://github.com/zhaoyanglab/ML-for-PSC-differentiation. All additional information will be made available upon reasonable request to the authors.

## Code availability

The code for training and testing the ML models is available in the GitHub repository https://github.com/zhaoyanglab/ML-for-PSC-differentiation.
