# Supplementary material for: A live-cell image-based machine learning strategy for reducing variability in PSC differentiation systems
Source: Cell Discov. 2023 Jun 6;9:53. doi: 10.1038/s41421-023-00543-1 (PMC10244346; doi:10.1038/s41421-023-00543-1)
Supplement: Supplementary file 1 — Supplementary information [file 41421_2023_543_MOESM1_ESM.pdf]

Supplementary Information

A live-cell image-based machine learning strategy for reducing variability in PSC differentiation systems

Xiaochun Yang, Daichao Chen, Qiushi Sun, Yao Wang, Yu Xia, Jinyu Yang, Chang Lin, Xin Dang, Zimu Cen, Dongdong Liang, Rong Wei, Ze Xu, Guangyin Xi, Gang Xue, Can Ye, Li-Peng Wang, Peng Zou, Shi-Qiang Wang, Pablo Rivera-Fuentes, Salome Püntener, Zhixing Chen, Yi Liu, Jue Zhang, Yang Zhao

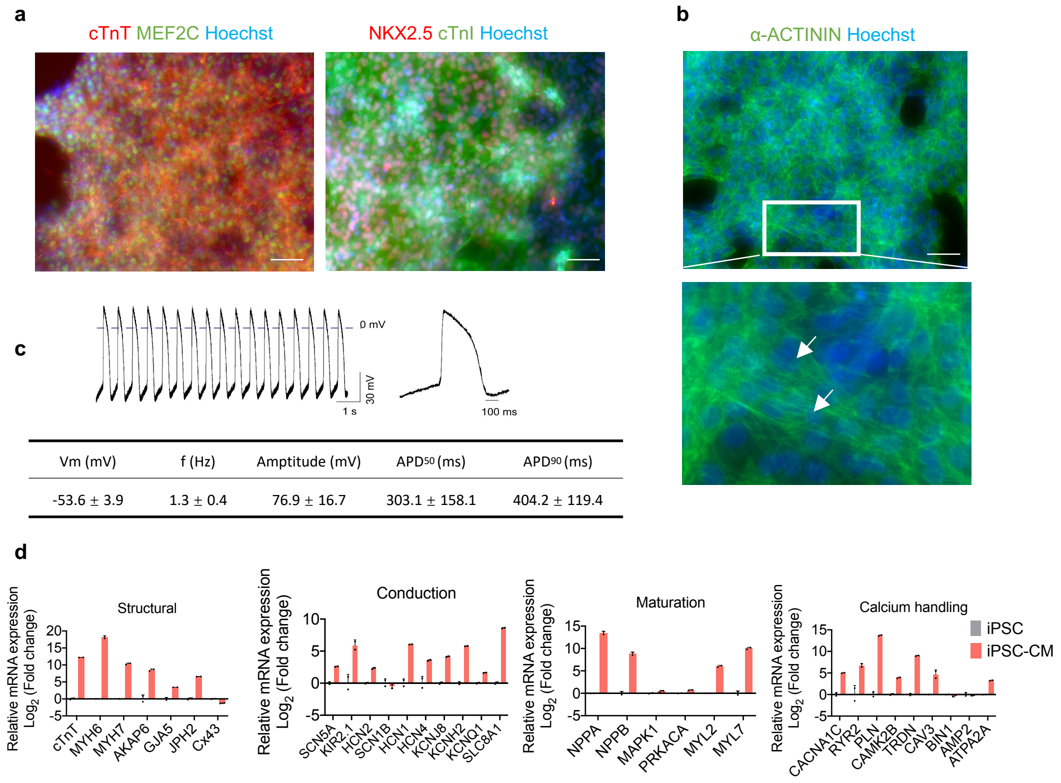

Supplementary Fig. S1 Characterization of PSC-CMs.

a Representative Immunofluorescent images of PSC-CM for cTnT, MEF2C, NKX2.5, and cTNI on day 12. Scale bar, 100 μm.

b Representative Immunofluorescent images of PSC-CM for α-ACTININ on day 12. Arrows show the sarcomere structures indicated by α-ACTININ staining. Scale bar, 100 μm.

c Representative recordings of spontaneous action potentials in single PSC-CMs on day 15. Summarized data on resting potential (Vm), frequency (f), action potential amplitude, and action potential duration at 50% of the amplitude (APD50) and 90% of the amplitude (APD90). Data were mean ± SD. *n* = 4.

d RT-PCR analysis of cardiac gene expression for PSC-CM (day 15, after purification) compared with PSC. *n* = 3.

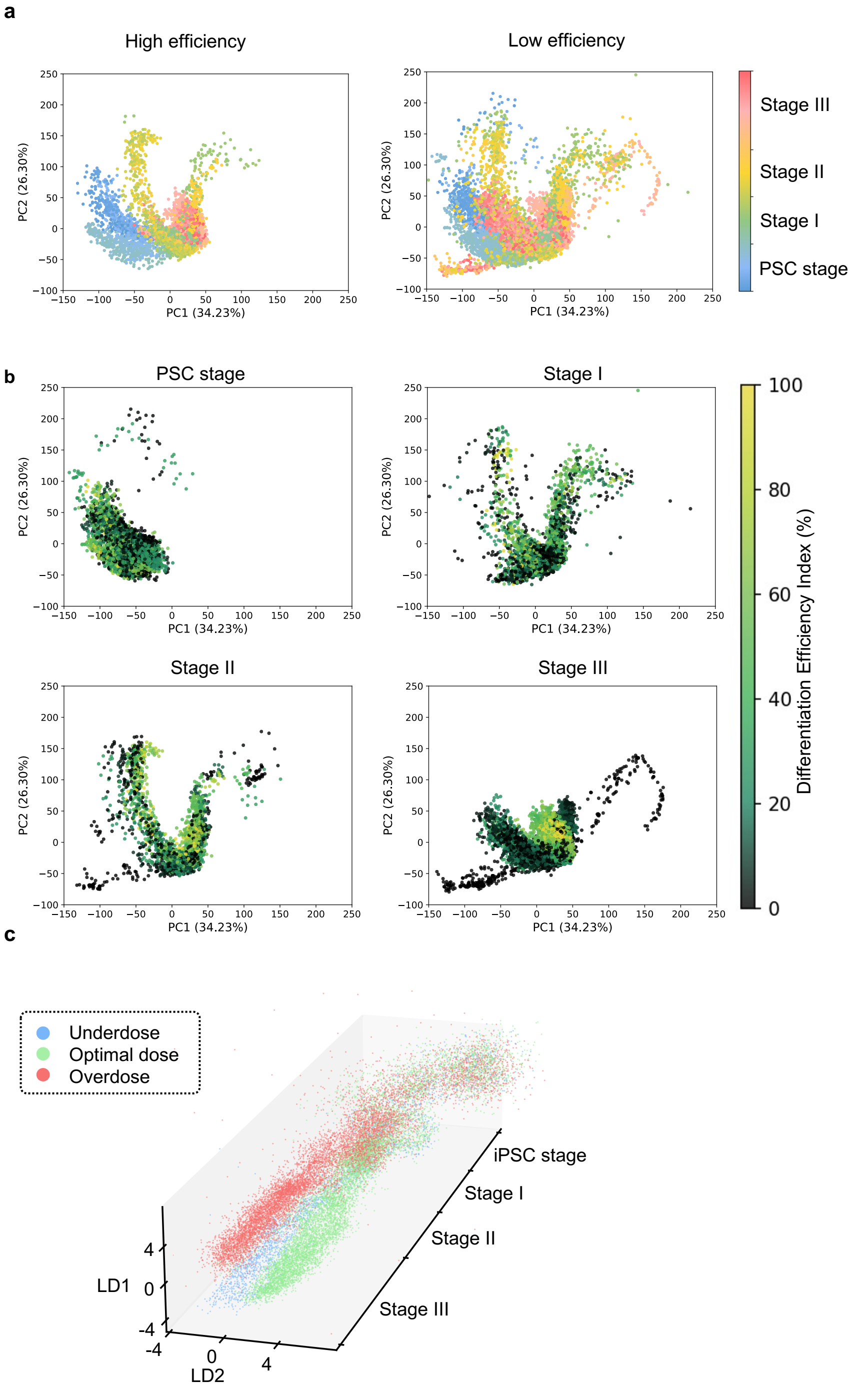

Supplementary Fig. S2 Visualization of local bright-field image features of the whole PSC-to-CM differentiation process.

a PCA of local bright-field image features of high-efficiency wells (Normalized Differentiation Efficiency Index ≥ 50%, n = 5200 images) and low-efficiency wells (Normalized Differentiation Efficiency Index < 50%, n = 14,768 images). The bright-field image of each well at a certain time point was represented by a 448-D texture feature vector and projected to the 2-D plane spanned by the first two principal components (PC1 and PC2). Colors represent the time points in the differentiation process. The Differentiation Efficiency Index was normalized between 0 and 100%.

b PCA of local bright-field image features at PSC stage, stage I, stage II, and stage III. The color of the dots represents the Differentiation Efficiency Index, which was normalized between 0 and 100%. n = 19,968 images.

c LDA of local bright-field image features. The axis of the 3-D plot represents the time axis and the first two linear discriminants (LD1 and LD2). n = 19,968 images.

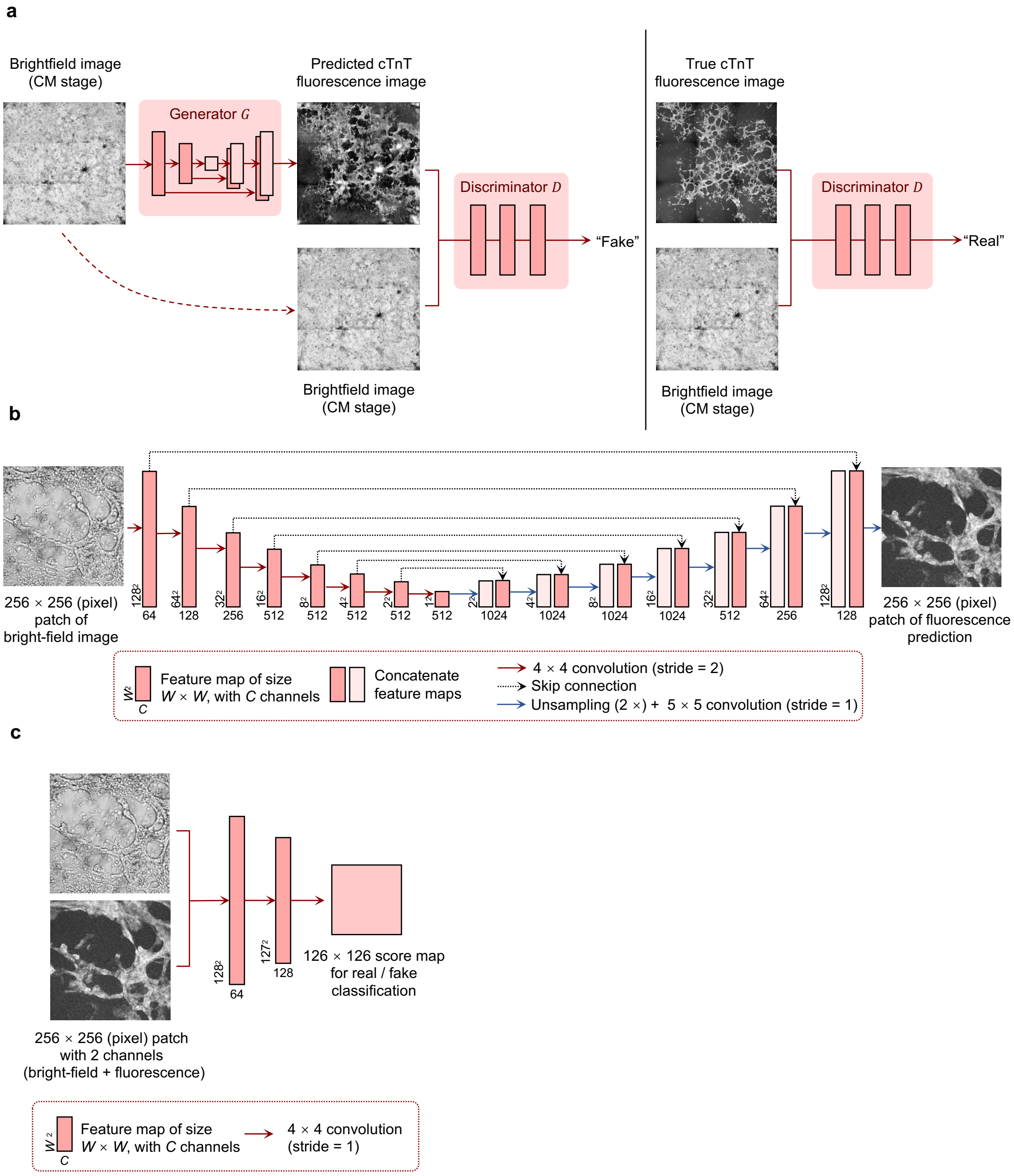

Supplementary Fig. S3 Schematic overview of deep learning-based cTnT fluorescence prediction on live-cell bright-field images at CM stage.

a Training the pix2pix model for brightfield-to-fluorescence image translation. The generator G learns to predict fluorescent labels from brightfield images, while the discriminator D learns to distinguish between true and fake "brightfield-fluorescence" image pairs.

b Detailed architecture of the generator. The generator G is a U-Net with 8 convolutional layers in both the encoder and decoder parts. All the inner convolutional layers are followed by Instance Normalization and ReLU activation. The transposed convolution in the original design was replaced with nearest neighbor up-sampling followed by 5 × 5 convolution.

c Detailed architecture of the discriminator. The discriminator D is a 3-layer convolutional neural network. Each pixel in the network output has a receptive field of size 16 × 16, representing the real/fake classification score for the corresponding 16 × 16 patches.

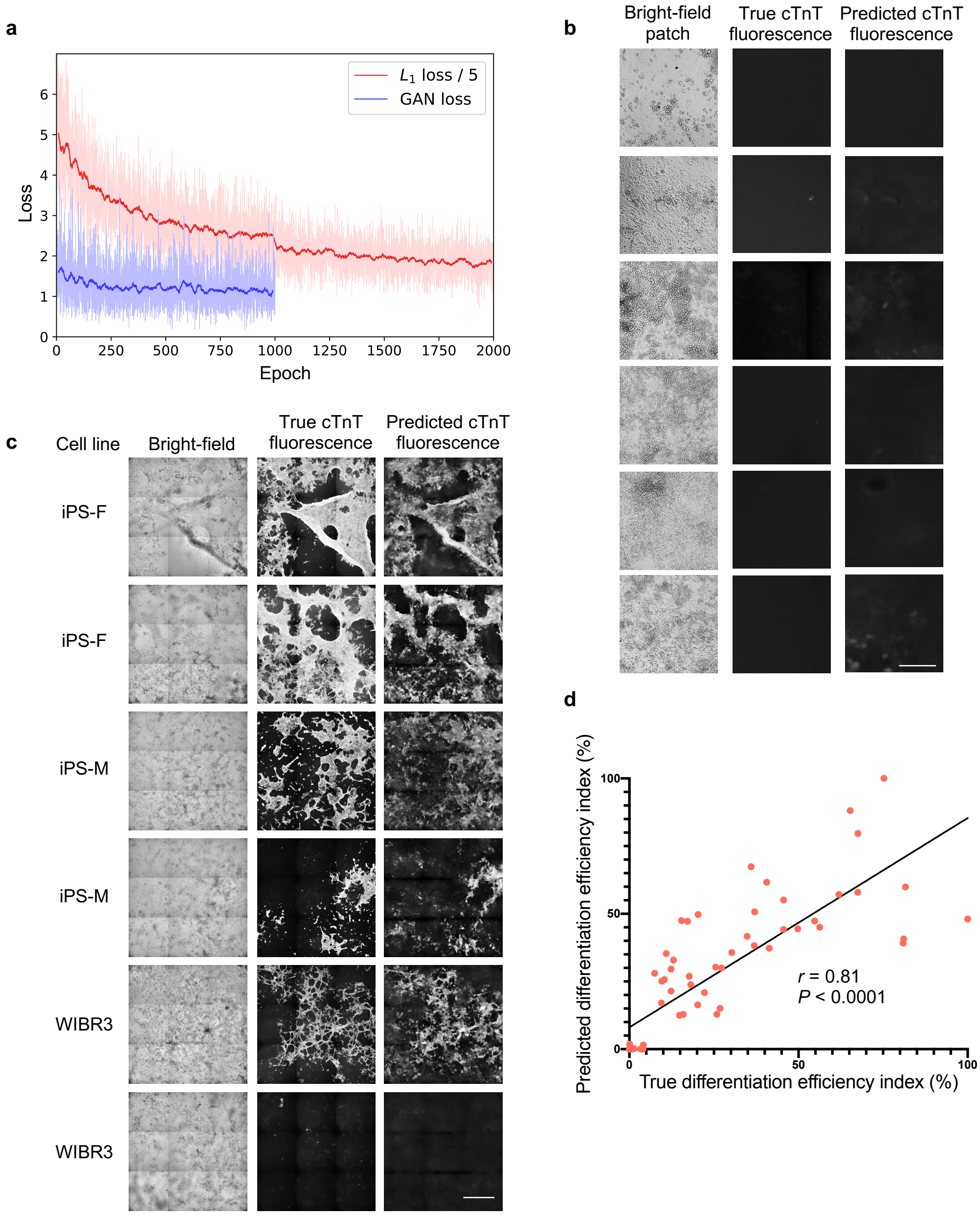

Supplementary Fig. S4 Performance of deep learning-based cTnT fluorescence prediction on live-cell bright-field images at CM stage.

a Change of L_1 loss and GAN loss of the U-Net generator in the pix2pix model during training. The L_1 loss is scaled by 1/5. The dark lines are the corresponding smoothed curves using 20-epoch moving average. The U-Net generator was trained with L_1 loss and GAN loss in the first 1000 epochs, while only trained with L_1 loss in the last 1000 epochs to ensure fidelity of fluorescence prediction.

b Typical prediction results of CM recognition on bright-field image patches (barely containing cTnT$^+$ CMs) from the test dataset. Each column from left to right represents: live-cell bright-field image patch; true cTnT fluorescence result; predicted cTnT fluorescence result. Scale bar, 250 µm.

c Typical prediction results of CM recognition on a new dataset from three additional cell lines (iPS-F, iPS-M, and WIBR3). Each column from left to right represents: live-cell bright-field image; true cTnT fluorescence result; predicted cTnT fluorescence result. Scale bar, 1 mm.

d Correlation between the true and predicted Differentiation Efficiency Indexes on a new dataset from three additional cell lines (iPS-F, iPS-M, and WIBR3), with Pearson's r value. True and predicted Differentiation Efficiency Indexes were normalized between 0 and 100%. n = 62 wells.

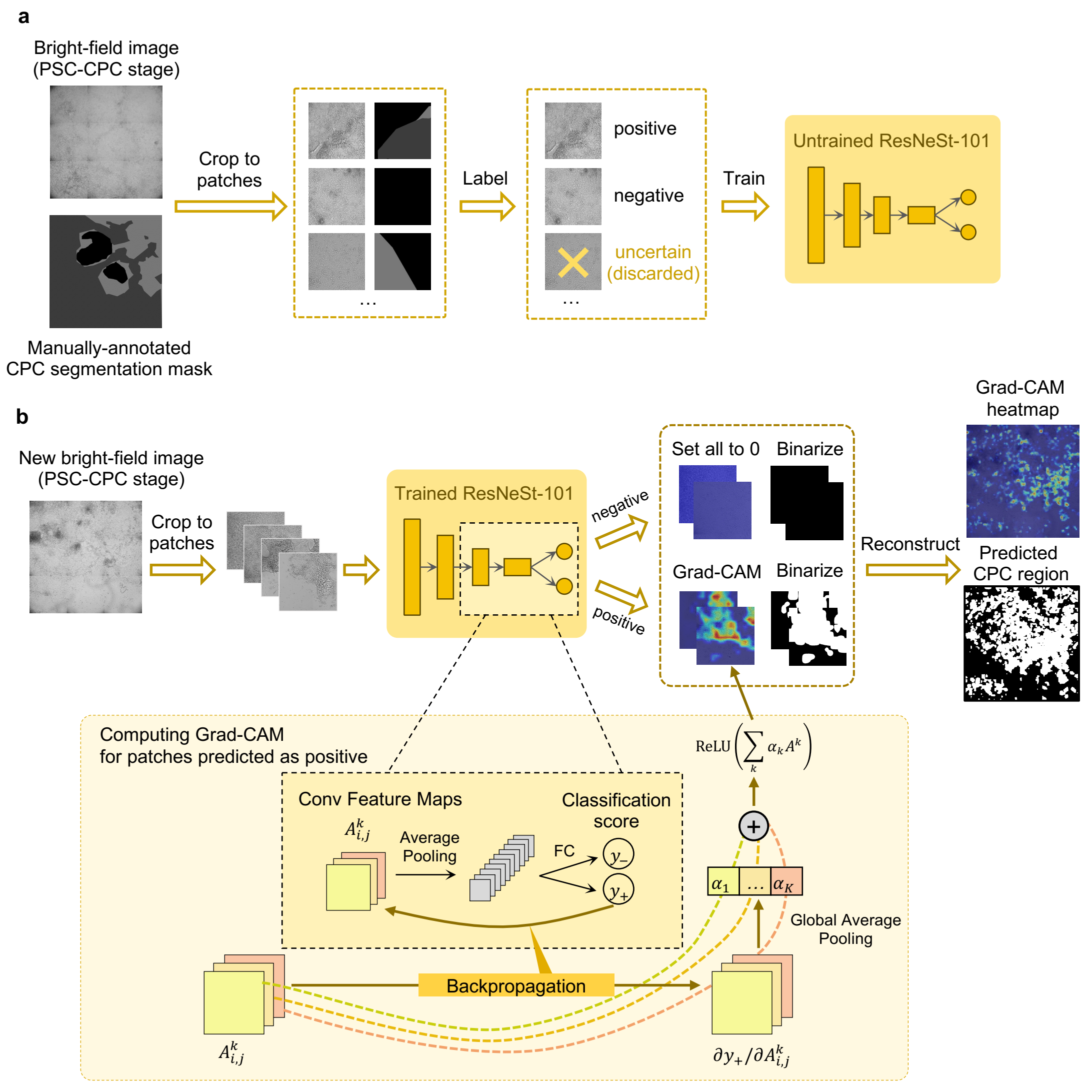

Supplementary Fig. S5 Schematic overview of training and testing of the weakly supervised learning for CM-committed CPC recognition.

a Schematic overview of the training stage. The ResNeSt-101 classification network was trained with bright-field patches labeled as positive or negative. The label of bright-field patches was derived from the manually annotated CPC segmentation masks, colored with dark grey, light gray, and black (Methods). The bright-field patches were labeled as “positive” or “negative”, or simply discarded according to the percentage of dark gray areas (Methods).

b Schematic overview of the test stage. Bright-field image patches were first classified using the trained ResNeSt: for patches predicted as “positive”, Grad-CAM was applied to generate a heatmap highlighting the regions important to the prediction; for patches predicted as “negative”, the heatmap was simply set to all-zero. Patch-level heatmaps and predicted CPC regions (by binarization) were finally reconstructed into whole-well predictions.

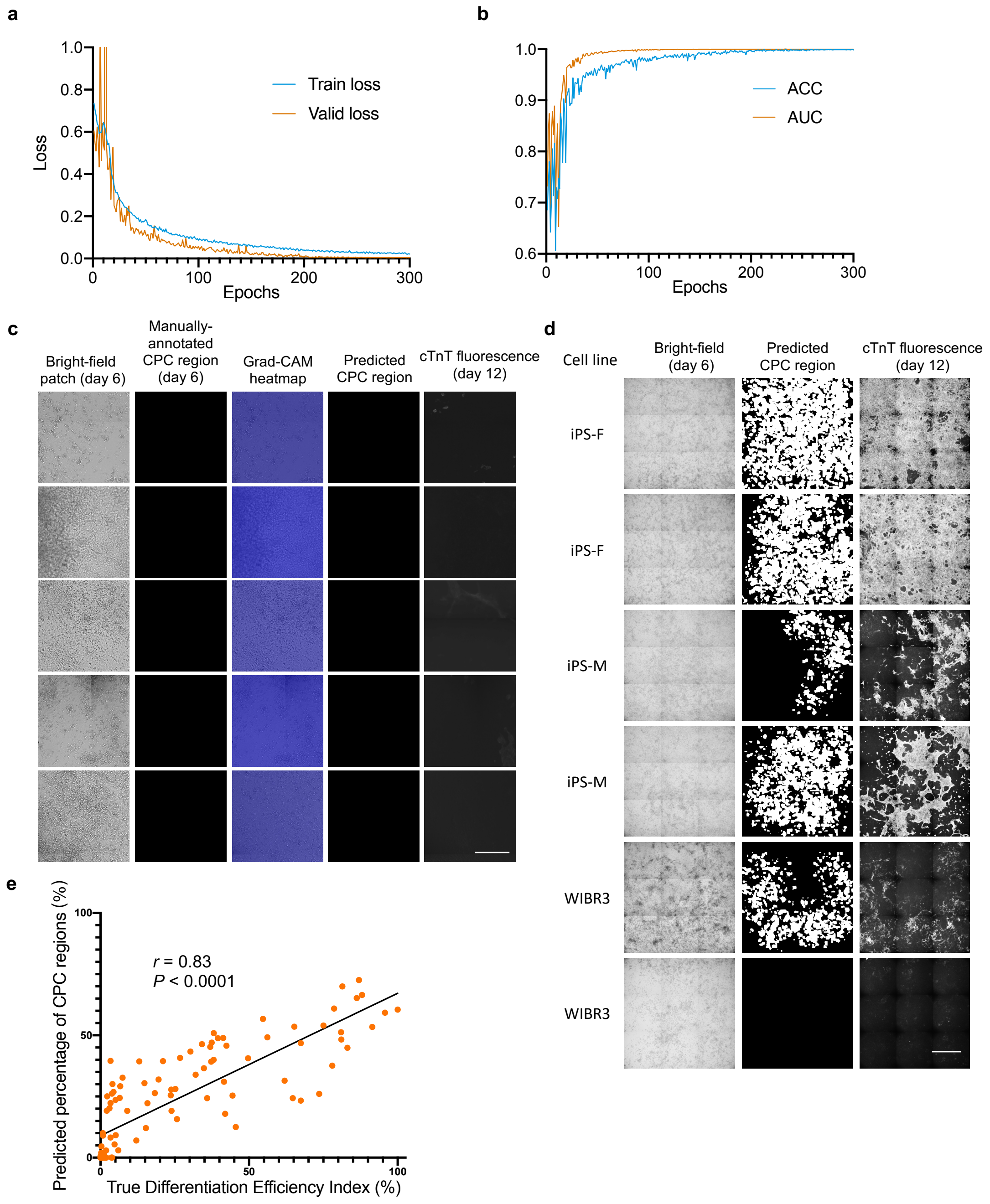

Supplementary Fig. S6 Performance of CPC recognition using the weakly supervised learning on images of CPC stage.

a Training loss and validation loss curves of the ResNeSt-101.

b Classification AUC and ACC curve of the ResNeSt-101.

c Typical prediction results of CPC recognition on the negative day 6 bright-field patches from the test set. Each column from left to right represents: live-cell bright-field image patch on day 6; manually annotated CPC regions; Grad-CAM heatmap for CPC localization; binary prediction result of CPC regions; true cTnT fluorescence result of day 12. Scale bar, 250 µm.

d Typical prediction results of CPC recognition on a new dataset from three additional cell lines (iPS-F, iPS-M, and WIBR3). Each column from left to right represents: live-cell bright-field image on day 6; binary prediction result of CPC regions; true cTnT fluorescence result of day 12. Scale bar, 1mm.

e Correlation between the true Differentiation Efficiency Index (from day 12 cTnT fluorescence results) and the predicted percentage of CPC regions (from day 6 CPC bright-field images) on a new dataset from three additional cell lines (iPS-F, iPS-M, and WIBR3), with Pearson's *r* value. True Differentiation Efficiency Index was normalized between 0 and 100%. $n = 103$ wells.

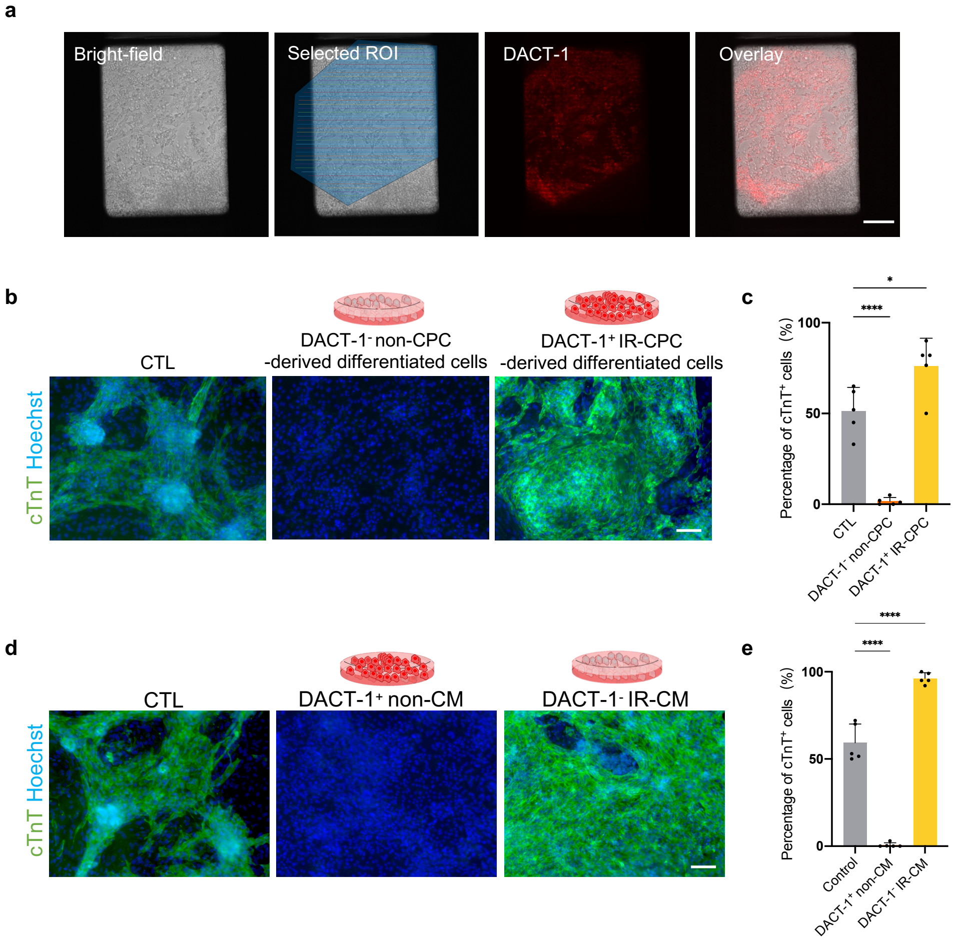

Supplementary Fig. S7 Purification of IR-CPC and IR-CM assisted by DACT-1 and laser scanning microscope.

a Effect of region-selective photoactivation using the ROI scanning mode. Images from left to right represent: live-cell bright-field image; selected ROI on bright-field for fluorescent labeling by 405 nm laser irradiating in which colored lines represent traces of violet light spot during ROI scanning; DACT-1-derived photoproduct in irradiated cell region detected in the 561 nm channel (red); the overlay of bright-field and 561 nm channel showing the positioning accuracy of fluorescent labeling. Scale bar, 100 µm.

b Purification effect of day 6 IR-CPCs. After FACS, unlabeled non-CPCs, DACT-1 labeled IR-CPCs, and cells of the CTL group were subsequently cultured for 6 days in RPMI + B27 medium. The purity of subsequently differentiated CMs was identified by immunostaining of cTnT. All the cells were from the same batch and under the same differentiation condition. Scale bar, 100 µm.

c Quantitative analysis of the percentage of $cTnT^{+}$ cells in (b). Data are mean ± SD. $n = 5$. * $P < 0.05$; **** $P < 0.0001$ by one-way ANOVA followed by Dunnett's multiple comparisons test.

d Purification effect of day 12 IR-CMs. After FACS, DACT-1 labeled non-CMs, unlabeled IR-CMs, and cells of the CTL group were subsequently cultured for 6 days in RPMI + B27 medium. The purity of CMs was identified by immunostaining of cTnT. All the cells were from the same batch under the same differentiation condition. Scale bar, 100 µm.

e Quantitative analysis of the percentage of $cTnT^{+}$ cells in (d). Data are mean ± SD. $n = 5$. **** $P < 0.0001$ by one-way ANOVA followed by Dunnett's multiple comparisons test.

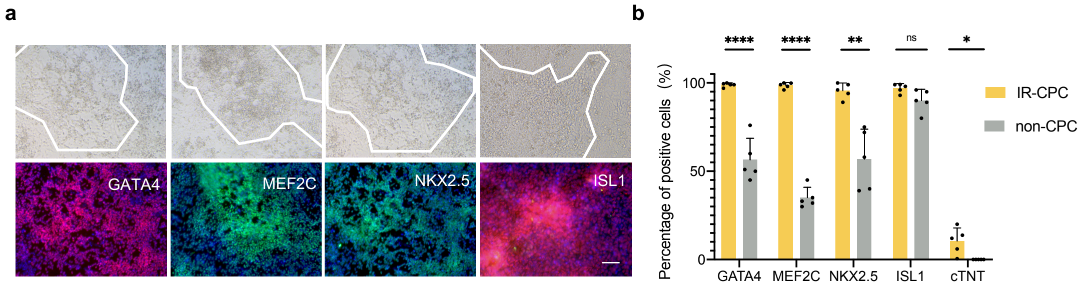

Supplementary Fig. S8 Characterization of IR-CPCs identified by immunofluorescence.

a Immunostaining of classical CPCs markers in the day 6 bright-field images. Cell regions with IR-CPC texture are in white frames. Scale bar, 100 µm.

b Quantitative analysis of CPC markers' distribution in IR-CPC or non-CPC region for (a). Data are mean ± SD. n = 5. Statistical significance was determined by t-test, * $P < 0.05$, ** $P < 0.01$, **** $P < 0.0001$.

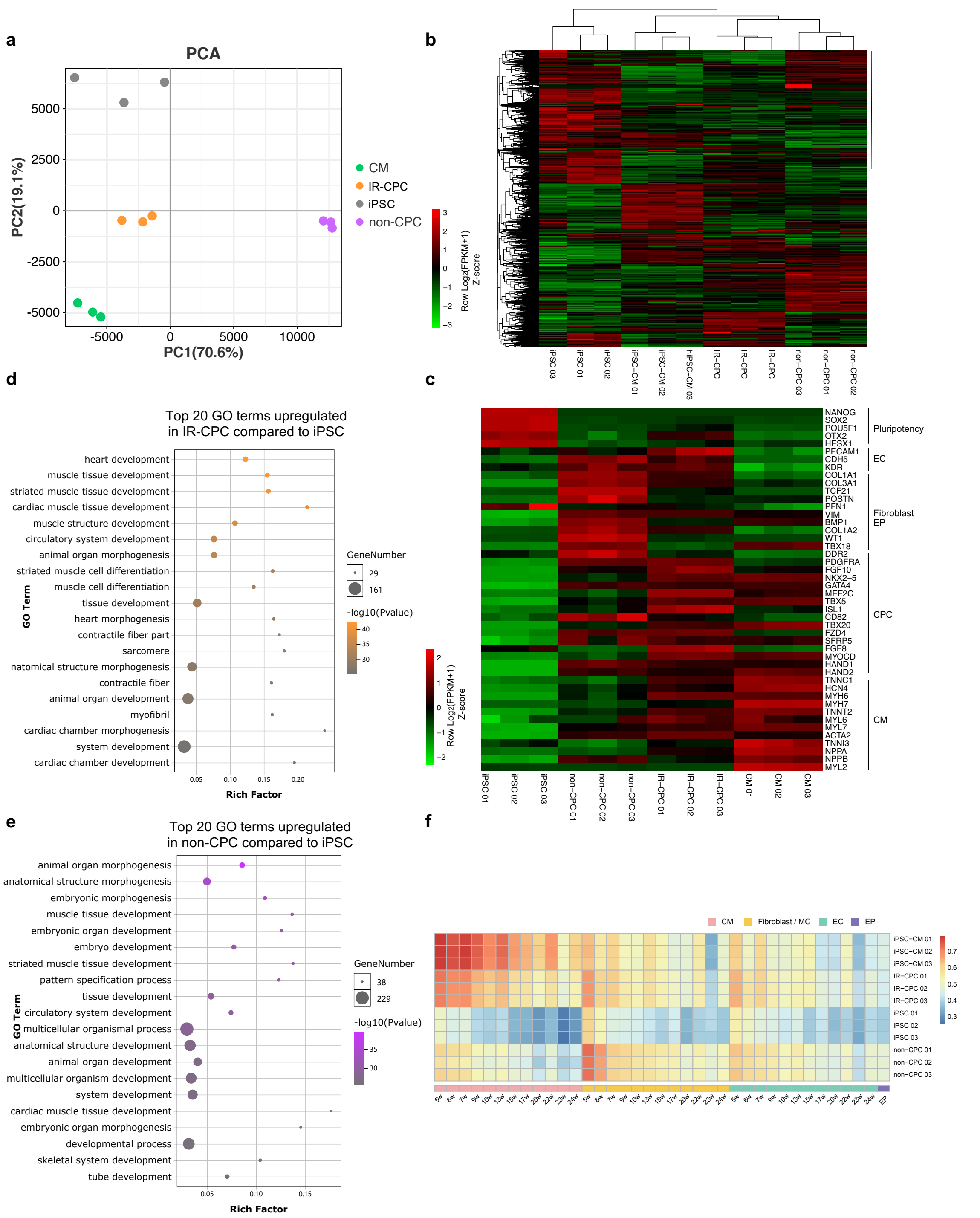

Supplementary Fig. S9 Characterization of IR-CPCs identified by bulk RNA-seq.

a PCA of bulk RNA-seq for iPSC, IR-CPC, non-CPC, and CM groups. Each point represents an RNA-seq sample. $n = 3$.

b Genome-wide comparison among iPSC, IR-CPC, non-CPC, and CM. Heatmap shows Z-scores of $\log_2$(FPKM+1) normalized over multiple samples. Hierarchical clustering was performed with 17,561 genes.

c Five separate gene clusters according to the different gene expression patterns in iPSC, IR-CPC, CM, and non-CPC. Bars on the right indicate identified lineages, including the pluripotent cell, endothelial cell (EC), fibroblast or epicardial cell (EP), CPC, and CM (from top to bottom). Heatmap shows Z-scores of $\log_2$(FPKM+1) normalized over multiple samples.

d Gene ontology (GO) enrichment analysis of DEGs on IR-CPC. The top 20 GO terms were enriched from the top 500 DEGs upregulated in IR-CPC compared to iPSC, most of which related to heart and cardiac muscle development.

e GO enrichment analysis of DEGs on non-CPC. Top 20 GO terms of top 500 genes upregulated in non-CPC compared to iPSC related to multiple tissue development.

f Comparison of cell types between single-cell RNA-seq data of human fetal heart development (column) and our bulk RNA-seq data (row). The correlation was calculated by applying the Spearman method. Four main cell types were retained for comparison: CM, fibroblast/mesenchymal cell (MC), EC, and EP. The week is denoted as "W". Color bar represents different cell types.

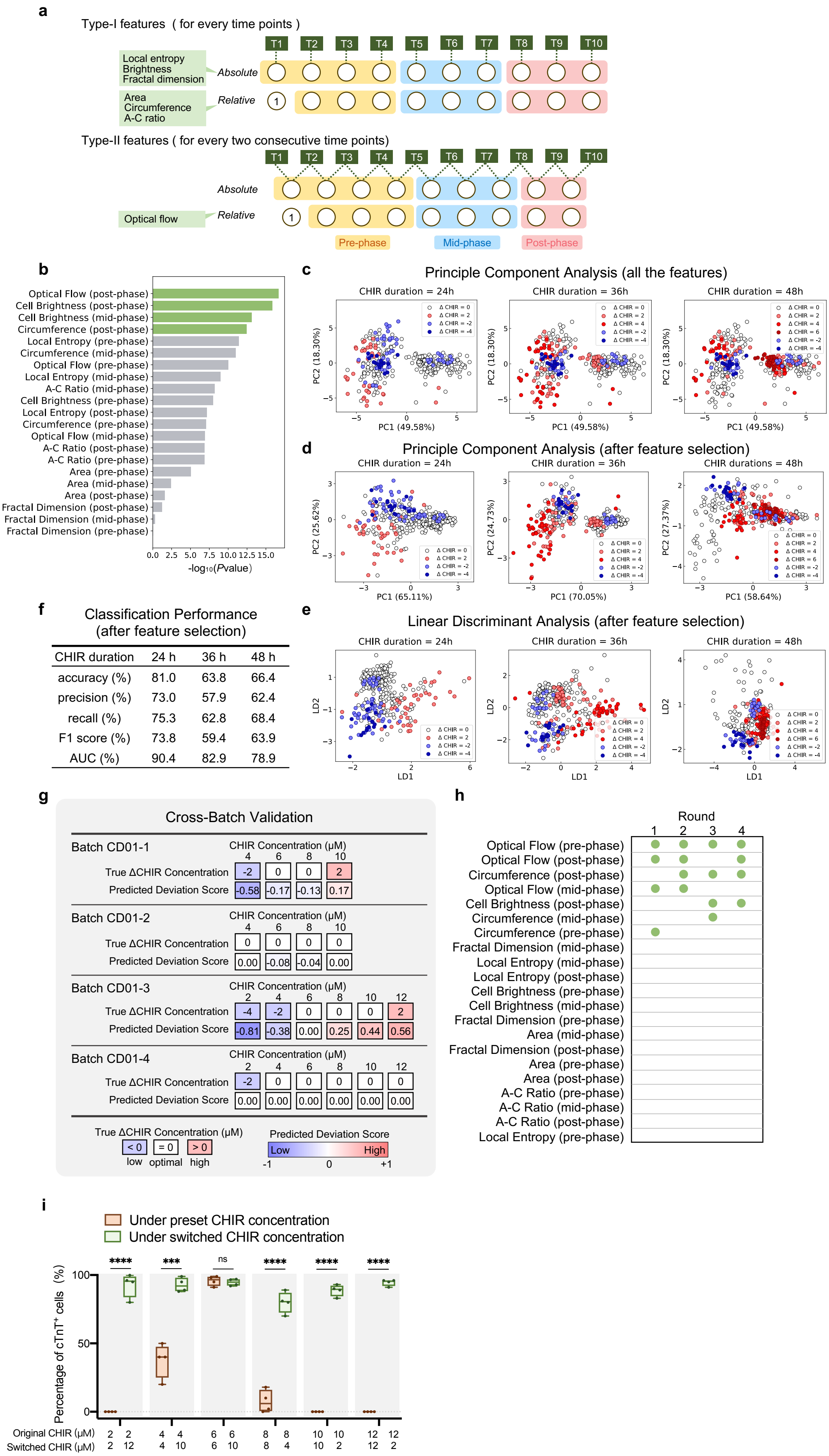
a
Type-I features (for every time points)
T1 T2 T3 T4 T5 T6 T7 T8 T9 T10
Local entropy
Brightness
Fractal dimension
Absolute
Area
Circumference
A-C ratio
Relative
1
Type-II features (for every two consecutive time points)
T1 T2 T3 T4 T5 T6 T7 T8 T9 T10
Absolute
Optical flow
Relative
1
Pre-phase
Mid-phase
Post-phase
b
Optical Flow (post-phase)
Cell Brightness (post-phase)
Cell Brightness (mid-phase)
Circumference (post-phase)
Local Entropy (pre-phase)
Circumference (mid-phase)
Optical Flow (pre-phase)
Local Entropy (mid-phase)
A-C Ratio (mid-phase)
Cell Brightness (pre-phase)
Local Entropy (post-phase)
Circumference (pre-phase)
Optical Flow (mid-phase)
A-C Ratio (post-phase)
A-C Ratio (pre-phase)
Area (pre-phase)
Area (mid-phase)
Area (post-phase)
Fractal Dimension (post-phase)
Fractal Dimension (mid-phase)
Fractal Dimension (pre-phase)
0.0 2.5 5.0 7.5 10.0 12.5 15.0
-log10(Pvalue)
c
Principle Component Analysis (all the features)
CHIR duration = 24h
CHIR duration = 36h
CHIR duration = 48h
PC1 (49.58%)
PC2 (18.30%)
d
Principle Component Analysis (after feature selection)
CHIR duration = 24h
CHIR duration = 36h
CHIR durations = 48h
PC1 (65.11%)
PC2 (25.62%)
PC1 (70.05%)
PC2 (24.73%)
PC1 (58.64%)
PC2 (27.37%)
f
Classification Performance
(after feature selection)
CHIR duration 24 h 36 h 48 h
accuracy (%) 81.0 63.8 66.4
precision (%) 73.0 57.9 62.4
recall (%) 75.3 62.8 68.4
F1 score (%) 73.8 59.4 63.9
AUC (%) 90.4 82.9 78.9
e
Linear Discriminant Analysis (after feature selection)
CHIR duration = 24h
CHIR duration = 36h
CHIR duration = 48h
LD1
LD2
g
Cross-Batch Validation
Batch CD01-1
CHIR Concentration (μM) 4 6 8 10
True ΔCHIR Concentration -2 0 0 2
Predicted Deviation Score -0.58 -0.17 -0.13 0.17
Batch CD01-2
CHIR Concentration (μM) 4 6 8 10
True ΔCHIR Concentration 0 0 0 0
Predicted Deviation Score 0.00 -0.08 -0.04 0.00
Batch CD01-3
CHIR Concentration (μM) 2 4 6 8 10 12
True ΔCHIR Concentration -4 -2 0 0 0 2
Predicted Deviation Score -0.81 -0.38 0.00 0.25 0.44 0.56
Batch CD01-4
CHIR Concentration (μM) 2 4 6 8 10 12
True ΔCHIR Concentration -2 0 0 0 0 0
Predicted Deviation Score 0.00 0.00 0.00 0.00 0.00 0.00
True ΔCHIR Concentration (μM)
< 0 = 0 > 0
low optimal high
Predicted Deviation Score
Low High
-1 0 +1
h
Round
1 2 3 4
Optical Flow (pre-phase)
Optical Flow (post-phase)
Circumference (post-phase)
Optical Flow (mid-phase)
Cell Brightness (post-phase)
Circumference (mid-phase)
Circumference (pre-phase)
Fractal Dimension (mid-phase)
Local Entropy (mid-phase)
Local Entropy (post-phase)
Cell Brightness (pre-phase)
Cell Brightness (mid-phase)
Fractal Dimension (pre-phase)
Area (mid-phase)
Fractal Dimension (post-phase)
Area (pre-phase)
Area (post-phase)
A-C Ratio (pre-phase)
A-C Ratio (mid-phase)
A-C Ratio (post-phase)
Local Entropy (pre-phase)
i
Under preset CHIR concentration
Under switched CHIR concentration
**** *** ns **** **** ****
Percentage of cTnT+ cells (%)
100
50
0
Original CHIR (μM) 2 2 4 4 6 6 8 8 10 10 12 12
Switched CHIR (μM) 2 12 4 10 6 10 8 4 10 2 12 2

Supplementary Fig. S10 Feature design and performance of assessment on the CHIR dose.

a Schematic of feature extraction. The 0–12 h image streams consisted of 10 uniformly-taken bright-field images (denoted by T1, T2, …, T10). The features are computed at every time point (for type-I features) or at every two consecutive time points (for type-II features). Relative features are normalized by T1 baselines. For each feature, the mean value in the pre-phase, mid-phase, and post-phase are used. Therefore, three numbers are obtained for each of the seven features, resulting in a 21-D feature representation.

b Importance weights of the 21 features. Wells were labeled as low, optimal, and high under a CHIR duration of 24 h. One-way ANOVA was conducted on the training set (n = 268 wells), yielding a Pvalue for each feature.

c PCA of 0–12h image streams represented by 21 features. n = 384 wells.

d PCA of 0–12h image streams represented by the four selected features. n = 384 wells.

e LDA of 0–12h image streams represented by the four selected features. n = 384 wells.

f Classification performance of the logistic regression model using the four selected features on the test set. n = 116 wells.

g Detailed results of the cross-batch validation (under a CHIR duration of 24 h). In each train-test round, one of the four batches was selected for testing while the other 3 batches were for training. The predicted Deviation Scores, with true CHIR concentration labels, were provided.

h Features selected in each round of cross-batch validation. Green dots represent the top four features with the most important weights in each round.

i Effects of switching CHIR concentration. For each well, the preset CHIR concentration was maintained or switched to a more suitable condition at 24 h. The percentage of $cTnT^+$ cells for wells under a preset CHIR concentration and under an optimized CHIR concentration was shown (CHIR duration = 48 h).

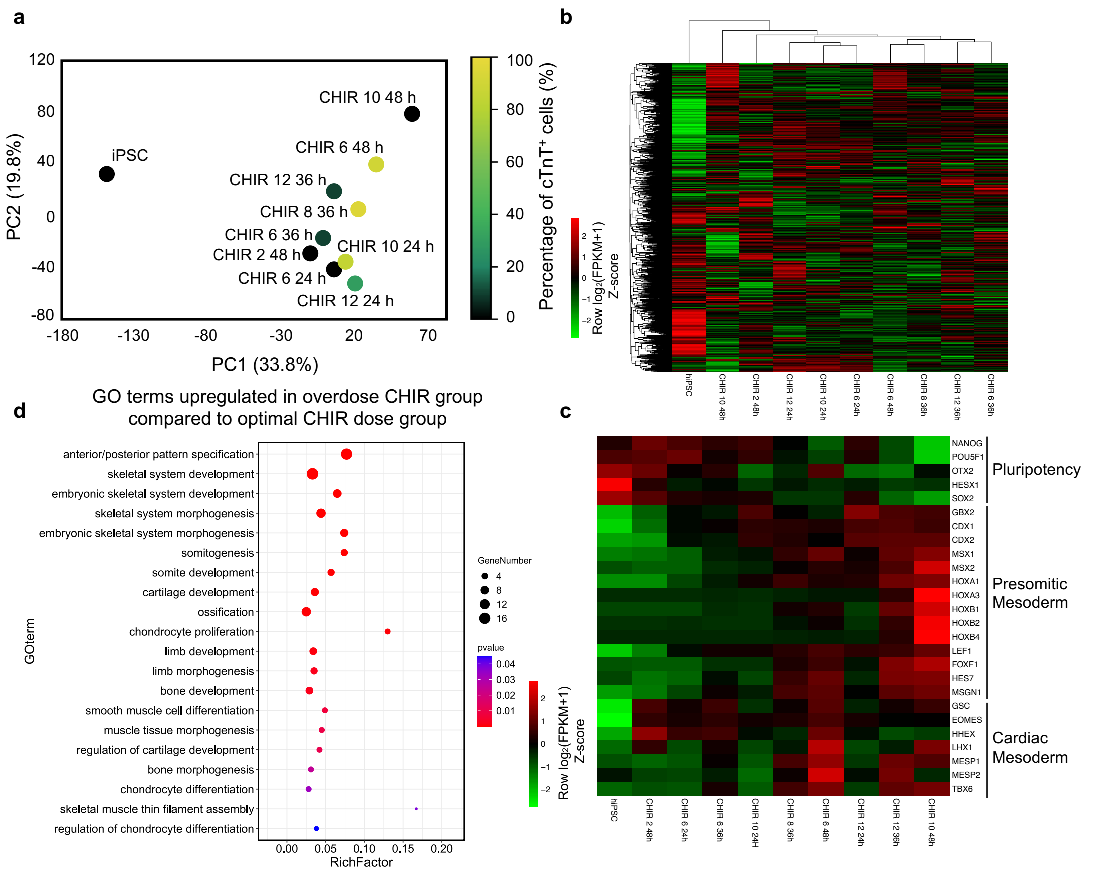

Supplementary Fig. S11 Identification of cell response to different CHIR doses by bulk RNA-seq.

a PCA of bulk RNA-seq for different CHIR doses. Sequencing samples were collected at stage I of differentiation. For each CHIR dose, average CM differentiation efficiency was quantified from the day 12 cTNT immunofluorescence results (*n* = 5). Sequencing samples and the samples staining for cTNT were from the same batch to avoid CHIR fluctuation.

b Genome-wide comparison among the samples treated with different CHIR doses. Heatmap shows Z-scores of log_2(FPKM+1) normalized over multiple samples. Hierarchical clustering was performed with 16,220 genes.

c Different mesodermal fates under different CHIR doses revealed by RNA-seq. Heatmap shows Z-scores of log_2(FPKM+1) normalized over multiple samples.

d GO enrichment analysis of up-regulated genes in overdose CHIR (10 μM 48 h) treatment compared with optimum CHIR dose (6 μM 48 h). 20 GO terms related to somitogenesis and anterior/posterior pattern specification were enriched.

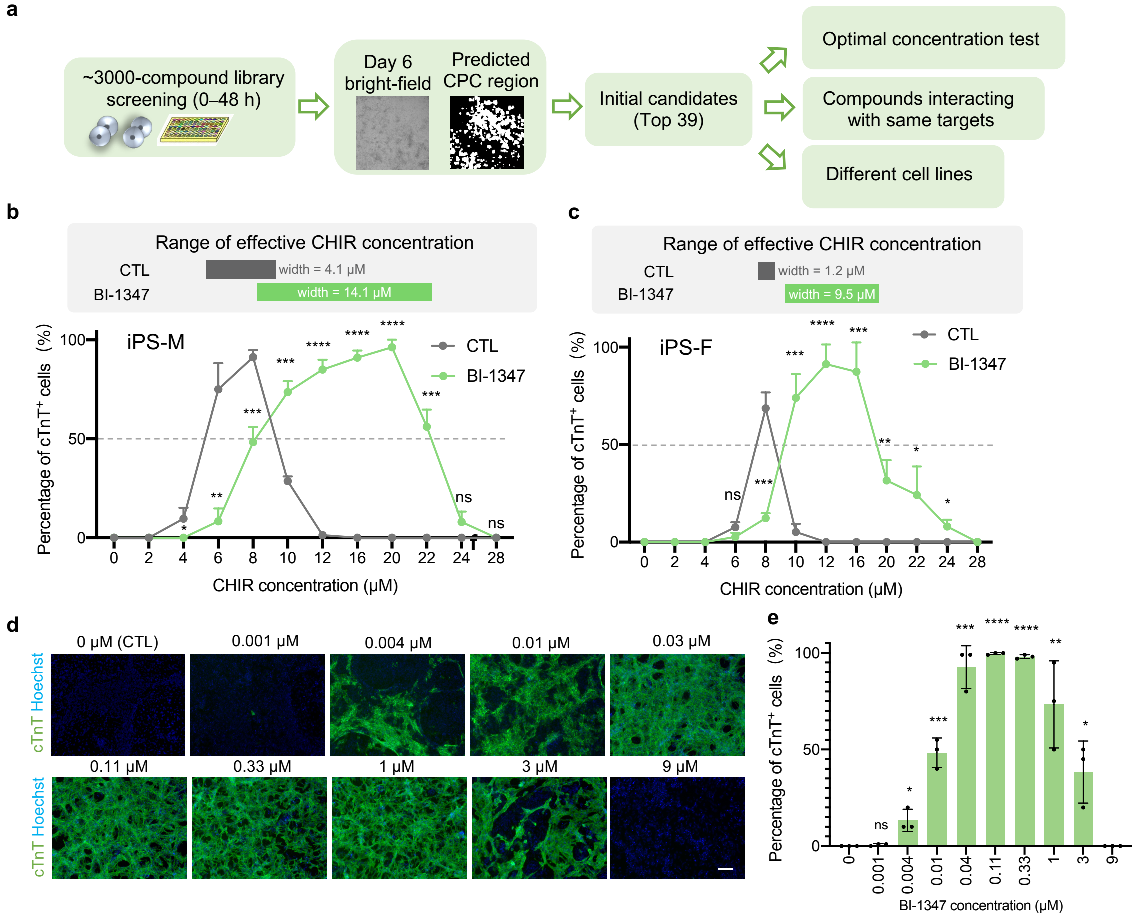

Supplementary Fig. S12 Image-based small molecule screening and verification of BI-1347.

a Workflow of the small molecule screening. iPS-B1 cell line was used for the preliminary screening.

b Effect of BI-1347 (0.5 μM, 48 h) on the iPS-M cell line. The widths of effective CHIR concentration (percentage of cTnT$^+$ cells ≥ 50%, indicates by a dash line) were shown in the upper panel. Data are mean ± SD. $n = 3$. Statistical significance was determined by *t*-test under each CHIR concentration. * $P < 0.05$, ** $P < 0.01$, *** $P < 0.001$, **** $P < 0.0001$; ns, not significant.

c Effect of BI-1347 (0.5 μM, 48 h) on the iPS-F cell line. The widths of effective CHIR concentration (percentage of cTnT$^+$ cells ≥ 50%, indicates by a dash line) were shown in the upper panel. Data are mean ± SD. $n = 3$. Statistical significance was determined by *t*-test under each CHIR concentration. * $P < 0.05$, ** $P < 0.01$, *** $P < 0.001$, **** $P < 0.0001$; ns, not significant.

d Effect of different concentrations of BI-1347 on improving differentiation efficiency under high CHIR dose (16 μM, 48 h). Representative immunofluorescent images of cTnT (green) and Hoechst (blue) on day 12 were shown. Scale bar, 100 μm.

e Effect of different BI-1347 concentrations on cardiac differentiation efficiency under high CHIR dose (16 μM, 48 h). iPSC was treated with BI-1347 during 0–48 h. Data are mean ± SD. $n = 3$. Statistical significance was determined by *t*-test to compare each BI-1347 concentration group with the CTL group (BI-1347 concentration = 0 μM). * $P < 0.05$, ** $P < 0.01$, *** $P < 0.001$, **** $P < 0.0001$; ns, not significant.

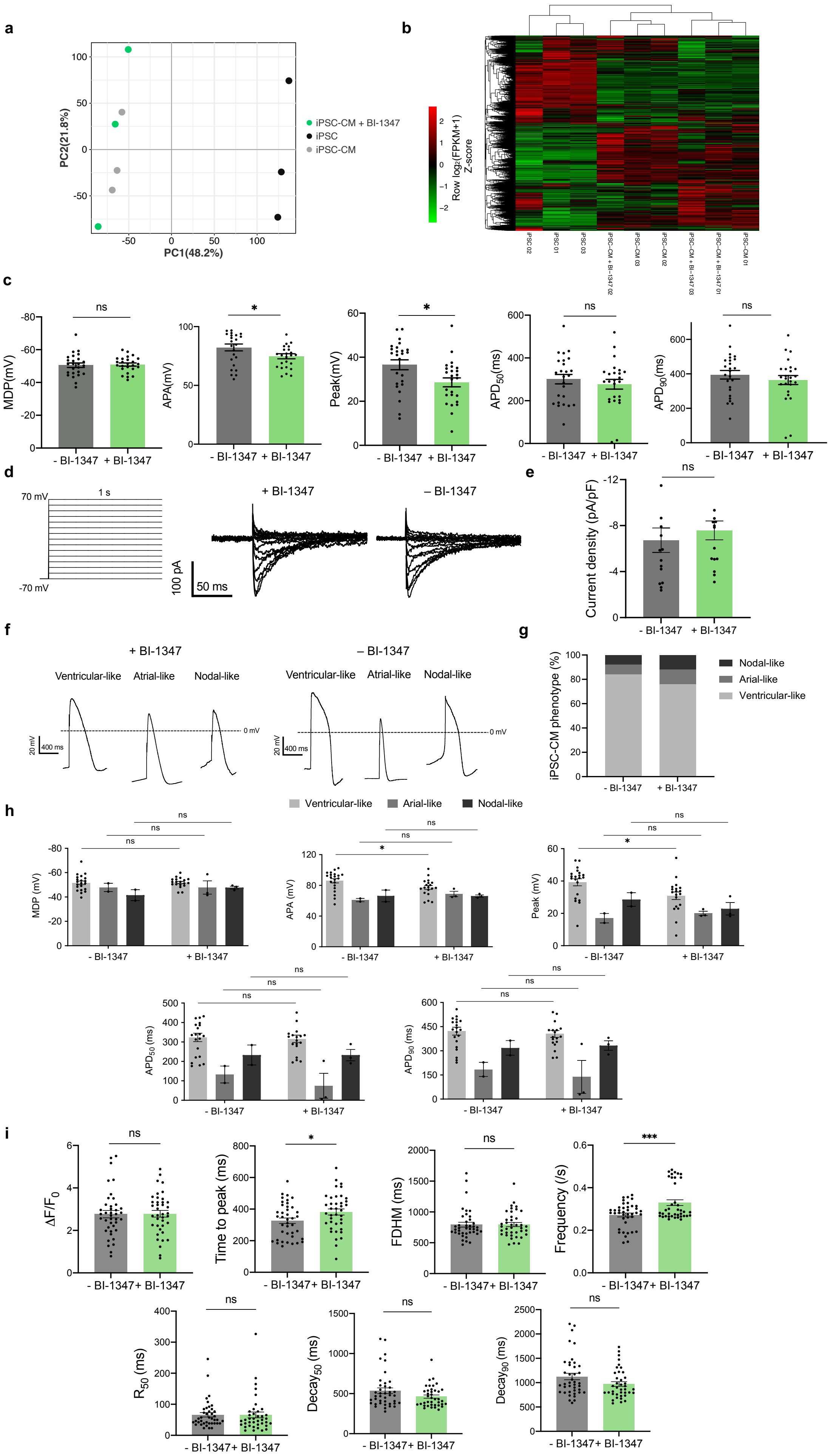

Supplementary Fig. S13 Characteristics of iPSC-CM treated with BI-1347.

a PCA of bulk RNA-seq for iPSC, iPSC-CM - BI-1347, and iPSC-CM + BI-1347 (0.5 μM, 48 h) groups. Each point represents an RNA-seq sample. *n* = 3.

b Genome-wide comparison among iPSC, iPSC-CM, and BI-1347 treated (0–48 h) iPSC-CM without metabolic purification. Heatmap shows Z-scores of log_2(FPKM+1) normalized over multiple samples. Hierarchical clustering was performed with 19,999 genes.

c Maximum diastolic potential (MDP), peak voltage, action potential amplitude (APA), and action potential duration (APD) at different levels of repolarization (90% and 50%) were compared between the + BI-1347 group (*n* = 25 cells) and the - BI-1347 group (*n* = 25 cells). Data were expressed as mean ± SEM. * $P < 0.05$; ns, not significant, determined by *t*-test.

d The protocol and representative calcium current recordings of voltage-clamp.

e Calcium current density of iPSC-CMs at -10 mV. Data were expressed as mean ± SEM. ns, not significant, determined by *t*-test.

f Representative action potential recordings of three iPSC-CM subtypes in + BI-1347 (*n* = 25 cells) and - BI-1347 (*n* = 25 cells) groups.

g Proportions of iPSC-CM subtypes.

h Maximum diastolic potential (MDP), peak voltage, action potential amplitude (APA), and action potential duration (APD) at different levels of repolarization (90% and 50%) were compared among the + BI-1347 ventricular-like group (*n* = 19 cells), the - BI-1347 ventricular-like group (*n* = 21 cells), the + BI-1347 atrial-like group (*n* = 3 cells), the - BI-1347 atrial-like group (*n* = 2 cells), the + BI-1347 nodal-like group (*n* = 3 cells), and the - BI-1347 nodal-like groups (*n* = 2 cells). Data were expressed as mean ± SEM. * $P < 0.05$ and ns, not significant, determined by *t*-test.

i $\Delta F/F_0$, time to peak, FDHM, frequency, time to 50% peak (R_{50}), time of Decay 50% ($Decay_{50}$), and time of Decay 90% ($Decay_{90}$) were compared between the + BI-1347 group (*n* = 39) and the - BI-1347 group (*n* = 40). Data were expressed as mean ± SEM. * $P < 0.05$, *** $P < 0.001$, and ns, not significant, determined by *t*-test.

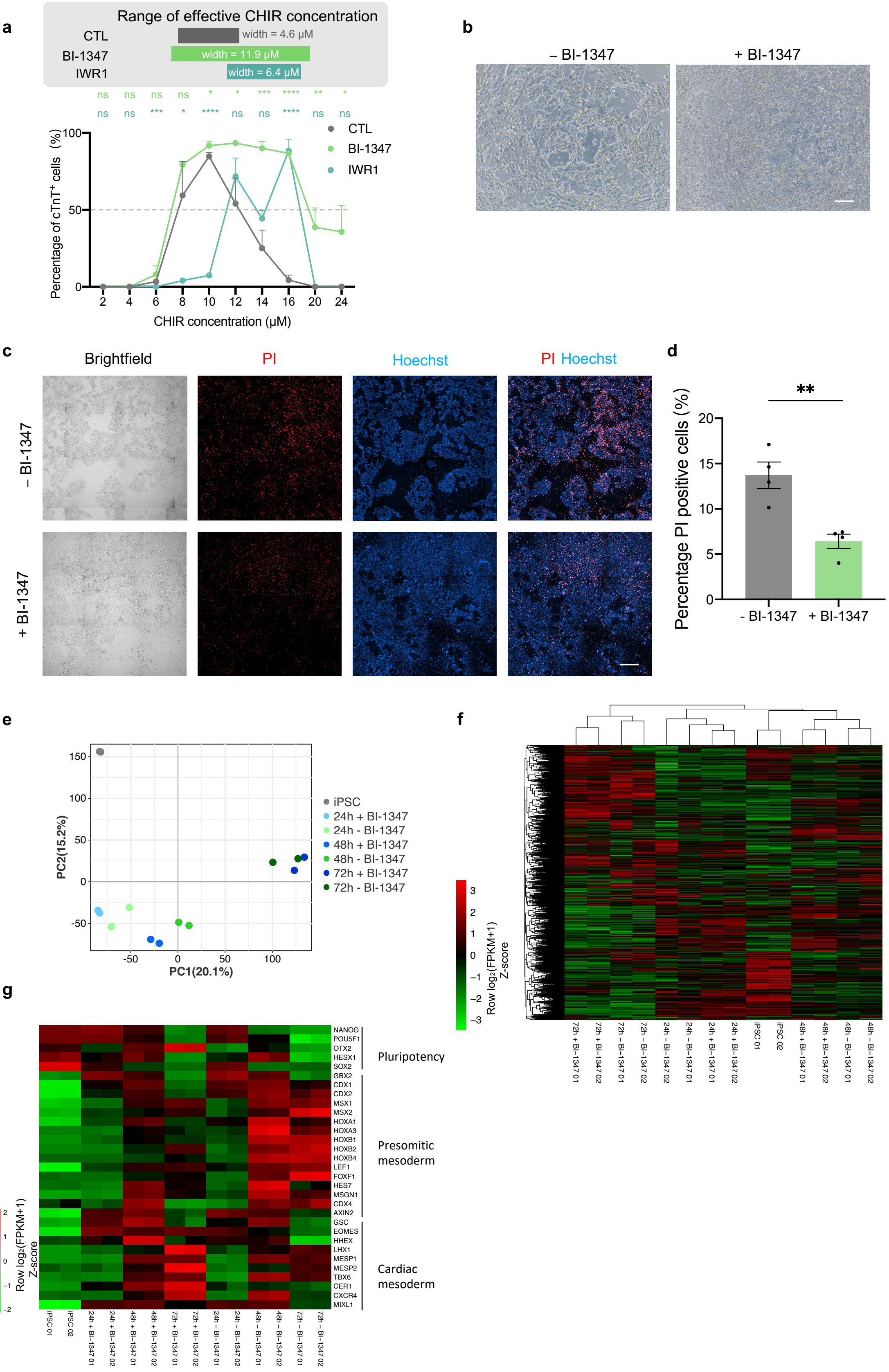

a
Range of effective CHIR concentration
CTL
width = 4.6 μM
BI-1347
width = 11.9 μM
IWR1
width = 6.4 μM
ns ns ns ns * * *** **** ** *
ns ns *** * **** ns ns **** ns ns
Percentage of cTnT+ cells (%)
100
50
0
2 4 6 8 10 12 14 16 20 24
CHIR concentration (μM)
CTL
BI-1347
IWR1
b
– BI-1347
+ BI-1347
c
Brightfield
PI
Hoechst
PI Hoechst
– BI-1347
+ BI-1347
d
**
Percentage PI positive cells (%)
20
15
10
5
0
- BI-1347
+ BI-1347
e
PC2(15.2%)
150
100
50
0
-50
-50
0
50
100
PC1(20.1%)
iPSC
24h + BI-1347
24h - BI-1347
48h + BI-1347
48h - BI-1347
72h + BI-1347
72h - BI-1347
f
3
2
1
0
−1
−2
−3
Row log2(FPKM+1)
Z-score
72h + BI-1347 01
72h + BI-1347 02
72h – BI-1347 01
72h – BI-1347 02
24h – BI-1347 02
24h – BI-1347 01
24h + BI-1347 01
24h + BI-1347 02
iPSC 01
iPSC 02
48h + BI-1347 01
48h + BI-1347 02
48h – BI-1347 01
48h – BI-1347 02
g
NANOG
POU5F1
OTX2
HESX1
SOX2
Pluripotency
GBX2
CDX1
CDX2
MSX1
MSX2
HOXA1
HOXA3
HOXB1
HOXB2
HOXB4
LEF1
FOXF1
HES7
MSGN1
CDX4
AXIN2
Presomitic
mesoderm
GSC
EOMES
HHEX
LHX1
MESP1
MESP2
TBX6
CER1
CXCR4
MIXL1
Cardiac
mesoderm
2
1
0
−1
−2
Row log2(FPKM+1)
Z-score
iPSC 01
iPSC 02
24h + BI-1347 01
24h + BI-1347 02
48h + BI-1347 01
48h + BI-1347 02
72h + BI-1347 01
72h + BI-1347 02
24h – BI-1347 01
24h – BI-1347 02
48h – BI-1347 01
48h – BI-1347 02
72h – BI-1347 01
72h – BI-1347 02

Supplementary Fig. S14 Effect of BI-1347 on inhibiting presomitic mesoderm and cell death under high CHIR dose.

a Effect of BI-1347 and IWR1 (a Wnt inhibitor) on differentiation efficiency in a wide range of CHIR concentrations. BI-1347 (0.5 μM, 48 h) or IWR1 (2 μM, 48 h) was added under different CHIR doses (from 4 to 24 μM, 48 h). The widths of effective CHIR concentration (percentage of cTnT+ cells ≥ 50%, indicates by a dash line) were shown in the upper panel. Data are mean ± SD. n = 3. Statistical significance was determined by t-test to compare the BI-1347 and IWR1 groups with the CTL group under each CHIR concentration. * $P < 0.05$, ** $P < 0.01$, *** $P < 0.001$, **** $P < 0.0001$; ns, not significant.

b Live-cell bright-field images at 48 h after CHIR treatment (12 μM, 48 h) with or without BI-1347 (0.5 μM, 48 h) treatment. Scare bar, 100 μm.

c Representative Hoechst / PI staining result for (a). Scare bar, 500 μm.

d Quantitative analysis of PI staining for (b). Data are mean ± SD. n = 4. Statistical significance was determined by t-test, ** $P < 0.01$.

e PCA of bulk RNA-seq for iPSC treated with or without BI-1347 (0.5 μM, 48 h) under the overdose CHIR (16 μM, 48 h). Samples were collected at different time points of stage I. Each point represents an RNA-seq sample. n = 3.

f Genome-wide comparison among the samples in (e). Heatmap shows Z-scores of $\log_2$(FPKM+1) normalized over multiple samples. Hierarchical clustering was performed with 19,999 genes.

g Mesodermal fate divergence under treatment with or without BI-1347 under the overdose CHIR (16 μM, 48 h) revealed by RNA-seq. Heatmap shows Z-scores of $\log_2$(FPKM+1) normalized over multiple samples.

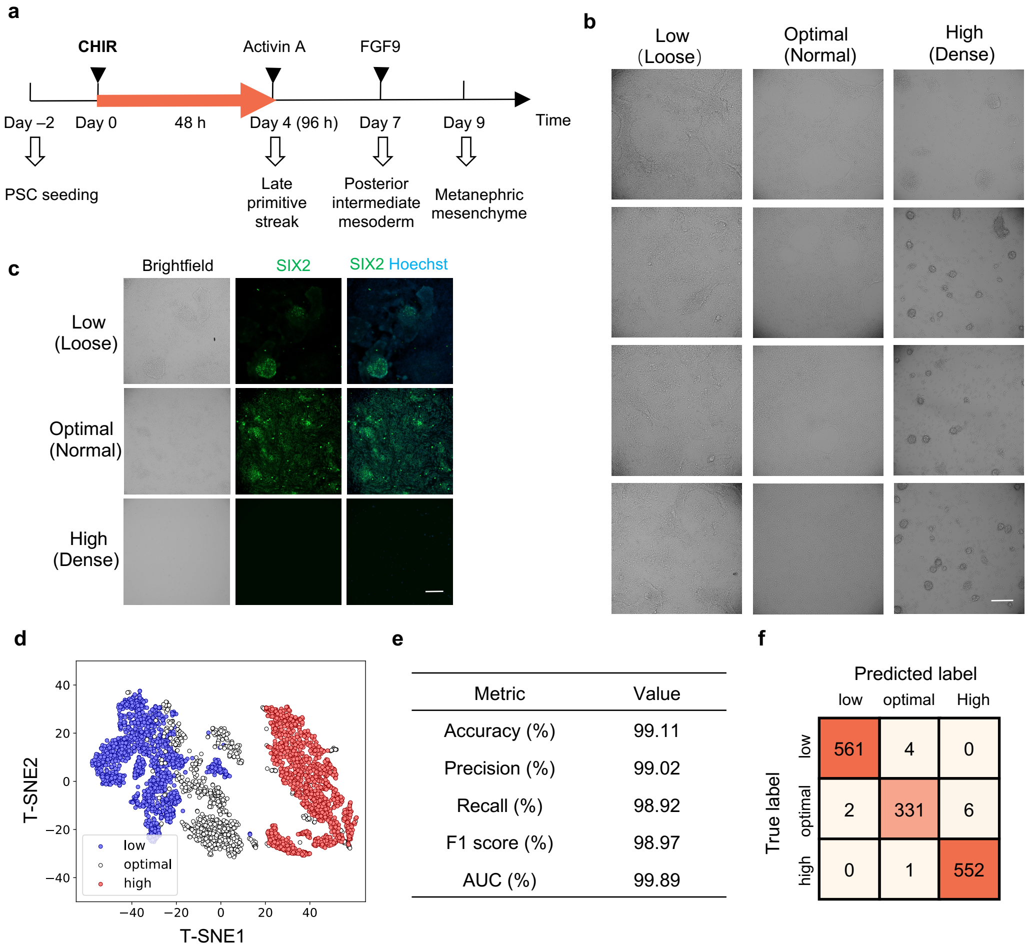

Supplementary Fig. S15 Early assessment of CHIR concentration in early nephric differentiation by ML.

a Schematic of PSC-to-NPC differentiation with CHIR as inducer. The red arrow indicates that different concentrations of CHIR used on day 0-4 could lead to different differentiation results. NPCs were collected on day 9 for SIX2 immunofluorescent staining.

b Typical bright-field images of day 4 cells under the treatment of low, optimal, and high CHIR concentrations. Scale bar, 200 µm.

c Representative SIX2 immunofluorescent images of NPCs under low, optimal, and high CHIR concentrations on day 9. Scale bar, 200 µm.

d T-SNE of the local features of day 4 bright-field images on the training set. $n = 3398$.

e Classification performance of the logistic regression model on the test set. Precision, recall, F1-score, and AUC were macro-averaged over the low, optimal, and high groups. $n = 1457$.

f Confusion matrix of the logistic regression model on the test set. $n = 1457$.

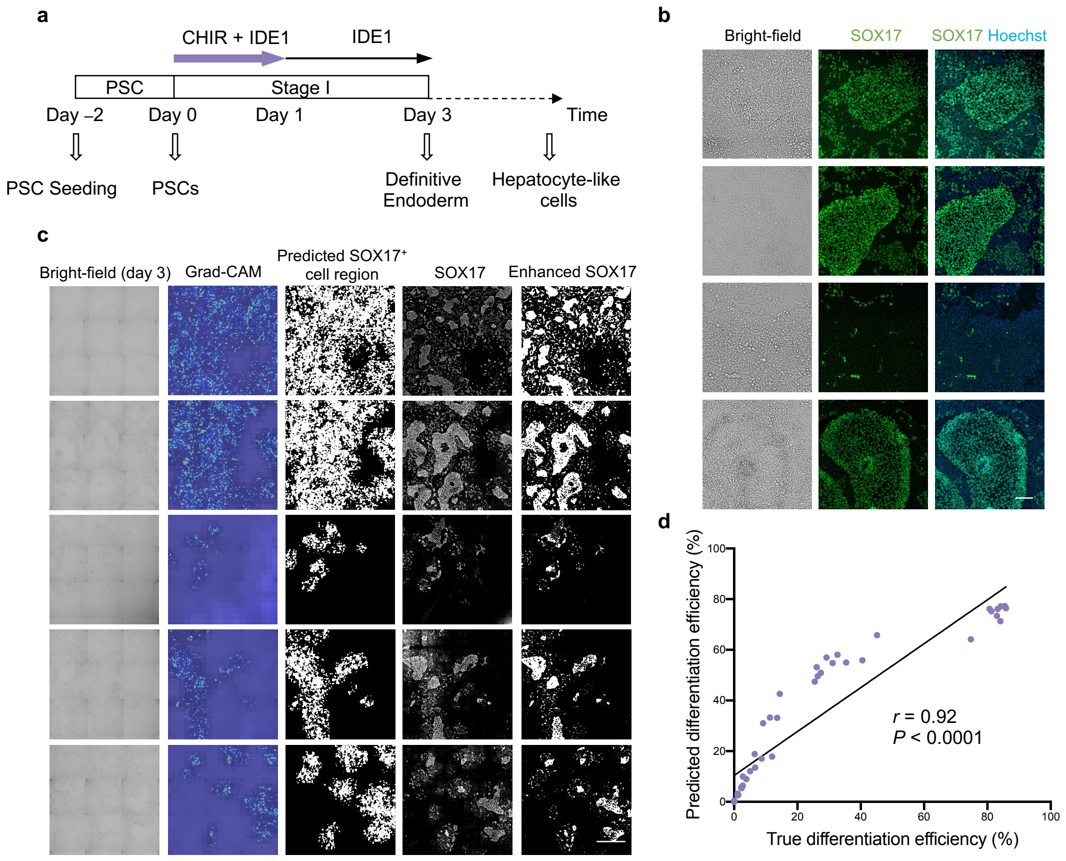

Supplementary Fig. S16 DE recognition for early hepatic differentiation by ML.

a Schematic of PSC-to-DE differentiation using small molecular for hepatocyte-like cell induction. DEs were collected on day 3 for SOX17 immunofluorescent staining.

b Representative Immunofluorescent results of SOX17 (green) and Hoechst (blue) for DE on day 3. Images with different final efficiencies (proportion of the $SOX17^+$ region) were chosen. Scale bar, 100 μm.

c Typical predicted results of DE recognition on bright-field images from the test set. Each column from left to right represents: live-cell bright-field image on day 3; Grad-CAM heatmap for endoderm cell localization; binary prediction for localization of $SOX17^+$ endoderm cell; true SOX17 fluorescence result of day 3; Enhanced fluorescence SOX17 images by binarization and morphological operations. Scale bar, 1 mm.

d Correlation between the true differentiation efficiency (from day 3 SOX17 fluorescence results) and the predicted differentiation efficiency (from day 3 bright-field images), with Pearson's r value. $n = 45$ images.

Supplementary Table S1 The dataset configuration of pix2pix model for PSC-CM recognition.

Batch No.	Cell lines	Medium	Day 0 iPSC/ESC Confluence	CHIR dose		Training data (wells)	Test data (wells)	Image size per well after preprocessing (pixels)	Training patch size (pixels)
				Concentration (μM)	Duration (h)				
CD03-1	iPS-18	RPMI+S12	~85%	2 4 6 8 10 12	18 24 30 36 42 48	9	7		
CD03-2	H9	RPMI+B27	~85%	2 4 6 8 10 12	18 24 30 36 42 54	5	4		
CD03-3	iPS-18	RPMI+B27	~85%	2 4 6 8 10 12	24 48	3	6		
CD03-4	iPS-18	RPMI+B27	~40–95%	4 6 8 10	24 30 36 42 48 54	10	13		
CD03-5	iPS-B1 H9	RPMI+B27	~70%	6 8 10 12 14 16	24 48	2	1	1536 × 1536	256 × 256
CD03-6	iPS-18	RPMI+B27	~85%	4 8 12	24 36 48	6	5		
CD03-7	iPS-F	RPMI+B27	~85%	4 6 8 10 12	48		31		
CD03-8	iPS-M	RPMI+B27	~85%	4 6 8 10 12	48		12		
CD03-9	WIBR3	RPMI+B27	~85%	4 6 8 10 12	48		19		

71 wells (from batch CD03-1 to CD03-6) were randomly divided into a training set (n = 35 wells) and a test set (n = 36 wells). 62 wells (from batch CD03-7 to CD03-9) from three additional cell lines were used for testing the trained model's generalization ability to new cell lines.

Supplementary Table S2 The dataset configuration of weakly supervised learning for PSC-CPC recognition.

Batch No.	Cell lines	Medium	CHIR dose		Day 0 iPSC/ESC Confluence	Training data			Test data				Image size per well after preprocessing (pixels)	Patch size (pixels)
			Concentration (μM)	Duration (h)		wells	Negative patches	Positive patches	wells	Negative patches	Positive patches	uncertain label		
CD02-1	iPS-18	RPMI+S12	2 4 6 8 10 12	18 24 30 36 42 48	~85%	23	961	760	6	187	863	1116		
CD02-2	iPS-18	RPMI+B27	4 6 8 10	24 30 36 42 48 54	~40–95%	46	1798	1615	17	1790	2382	1965		
CD02-3	iPS-18	RPMI+B27	2 4 6 8 10 12	24 48	~85%	7	22	625	2	83	547	92		
CD02-4	H9	RPMI+B27	2 4 6 8 10 12	18 24 30 36 42 54	~85%	15	342	908	4	181	1013	250		
CD02-5	iPS-B1 H9	RPMI+B27	6 8 10 12 14 16	24 48	~70%	12	52	1101	4	0	1395	49	2816 × 2816	512 × 512
CD02-6	iPS-B1	RPMI+B27	4 6 8 10	24 48	~85%	3	0	279	2	26	639	57		
CD02-7	WIBR3	RPMI+B27	4 6 8 10 12	48	~85%				19					
CD02-8	iPS-F	RPMI+B27	4 6 8 10 12	48	~85%				66					
CD02-9	iPS-M	RPMI+B27	4 6 8 10 12	48	~85%				41					

126 wells (from batch CD02-1 to CD02-6) were randomly divided into a training set (*n* = 106 wells) and a test set (*n* = 35 wells). The number of negative patches, positive patches, and uncertain patches was derived from the manually labeled CPC segmentation masks (see Methods). 126 wells (from batch CD02-7 to CD02-9) from three additional cell lines were used for testing the trained model's generalization ability to new cell lines. Manually labeled CPC segmentation masks were not available for batch CD02-7, CD02-8, and CD02-9.

Supplementary Table S3 The dataset configuration of ML for early assessment of CHIR doses.

Batch No.	Cell lines	Medium	Day 0 iPSC/ESC Confluence	CHIR duration (h)	24						36						48						Training data (wells)	Test data (wells)	Image size per well after preprocessing (pixels)
				CHIR concentration (μM)	2	4	6	8	10	12	2	4	6	8	10	12	2	4	6	8	10	12			
CD01-1	iPS-18	RPMI+B27	~40–95%	Mean percentage of cTnT⁺ cells (%)	-	7.8	45.0	40.3	6.8	-	-	47.5	83.8	10.5	0.3	-	-	37.5	82.5	0.8	0.5	-	66	30	4860 × 4860
CD01-2	iPS-B1	RPMI+B27	~50–70%		-	74.9	60.0	56.3	38.4	-	-	75.0	46.9	23.1	16.6	-	-	45.0	8.3	5.0	0.6	-	61	35	
CD01-3	H9	RPMI+B27	~85%		0.0	2.0	85.0	81.3	27.5	8.8	0.5	0.5	71.5	89.0	17.5	17.5	0.0	0.0	85.0	90.0	60.0	20.0	68	28	
CD01-4	iPS-B1	RPMI+B27	~60–95%		0.0	30.0	93.0	96.0	91.2	93.2	0.0	72.5	95.0	91.3	90.8	85.8	0.3	94.7	92.5	96.0	42.3	9.2	73	23	

384 wells (from batch CD01-1 to CD01-4) were randomly divided into a training set (*n* = 268 wells) and a test set (*n* = 116 wells). For each batch, CHIR concentrations with a mean percentage of $cTnT^+$ cells ≥ 20% were labeled as optimal, while concentrations out of the optimal concentration range were labeled as low or high.

Supplementary Table S4 The dataset configuration of ML for control of the initial PSC colony states.

Batch No.	Cell lines	Medium	CHIR dose		Training data (wells)	Test data (wells)	Image size per well after preprocessing (pixels)
			CHIR concentration (μM)	CHIR duration (h)			
CD00-1	iPS-B1	RPMI+B27	10	24	133	57	2151 × 2151
CD00-2	H9	RPMI+B27	6, 8	48	133	57	2231 × 2231
CD00-3	iPS-B1	RPMI+B27	16, 20	48	247	106	
CD00-4	iPS-B1	RPMI+B27	10, 12	48	131	57	
CD00-5	WIBR3	RPMI+B27	12, 14	48	134	58	
CD00-6	H9	RPMI+B27	12, 14	48	132	57	
CD00-7	iPS-F	RPMI+B27	6, 8	48	131	57	
CD00-8	iPS-M	RPMI+B27	6	48	44	20	
CD00-9	iPS-18	RPMI+B27	8, 10	48	132	57	
CD00-10	iPS-B1	RPMI+B27	6	48	133	58	

1934 wells (from batch CD00-1 to CD00-10) were randomly divided into a training set (n = 1350 wells) and a test set (n = 584 wells). To study the relationship between PSC colony morphology and the induction capacity, a wide range of duration of PSC stage (~2–5d) and day 0 confluence (~20–100%) were intentionally introduced.

Supplementary Table S5 Antibody list for immunofluorescence.

Antibody	Dilution	Company	Catalog Number
Rabbit polyclonal IgG anti-Mef2C	1:100	Thermo	PA5-82195
Rabbit polyclonal IgG anti-Islet1	1:500	Abcam	ab20670
Mouse monoclonal IgG1 anti-Mesp1	1:500	Novus Biologicals	NBP1-51613
Goat polyclonal IgG anti-cTnI	1:100	Abcam	ab56357
Sheep polyclonal IgG anti-Mef2c	1:100	R&D Systems	AF6786
Rabbit polyclonal IgG anti-Nkx2.5	1:200	Abcam	ab97355
Goat polyclonal IgG anti-Hand2	1:100	Novas;R&D	AF3876
Mouse monoclonal IgG2aκ anti-GATA-4	1:100	Santa Cruz Biotechnology	sc-25310
Mouse monoclonal IgG1 anti-cTnT	1:300	Thermo	MA5-12960
Rabbit polyclonal IgG anti-SIX2	1:200	Proteintech	11562-1-AP
Goat polyclonal IgG anti-SOX17	1:500	R&D Systems	AF1924
Rhodamine (TRITC) AffiniPure Donkey Anti-Rabbit IgG (H+L)	1:100	Jackson ImmunoResearch	711-025-152
Fluorescein (FITC) AffiniPure Donkey Anti-Rabbit IgG (H+L)	1:100	Jackson ImmunoResearch	711-095-152
Fluorescein (FITC) AffiniPure Donkey Anti-Goat IgG (H+L)	1:100	Jackson ImmunoResearch	705-095-147
Rhodamine (TRITC) AffiniPure Donkey Anti-Goat IgG (H+L)	1:100	Jackson ImmunoResearch	705-025-147
Rhodamine (TRITC) AffiniPure Donkey Anti-Mouse IgG (H+L)	1:100	Jackson ImmunoResearch	715-025-150
Fluorescein (FITC) AffiniPure Donkey Anti-Mouse IgG (H+L)	1:100	Jackson ImmunoResearch	715-095-150

Supplementary Table S6 Primers for RT-PCR.

Gene	Forward primer	Reverse primer
cTNT	ACAGAGCGGAAAAGTGGGAAG	TCGTTGATCCTGTTTCGGAGA
Cx43	GGTGACTGGAGCGCCTTAG	GCGCACATGAGAGATTGGGA
MYH6	GATAGAGAGACTCCTGCGGC	CCGTCTTCCCATTCTCGGTT
MYH7	TCGTGCCTGATGACAAACAGGAGT	ATACTCGGTCTCGGCAGTGACTTT
AKAP6	AGTTCTCCCTAAAGCTGCTGT	TCTGCCTAGTGTAGTTGCCATT
GJA5	AGAGTGTGAAGAAGCCCACG	AACAGATGCCAAAACTTCTGCT
JPH2	ACTCTGGCTCCTGGAACTTTG	GCGCCCCTTGGTCTCTATG
SLC8A1	TCATAGCTGATCGGTTCATGTCC	CAGTTGTCTTGGTGGTCTCTC
ATPA2A	CATCAAGCACACTGATCCCGT	CCACTCCCATAGCTTTCCCAG
CACNA1C	TGATTCCAACGCCACCAATTC	GAGGAGTCCATAGGCGATTACT
RYR2	CATCGAACACTCCTCTACGGA	GGACACGCTAACTAAGATGAGGT
PLN	ACCTCACTCGCTCAGCTATAA	CATCACGATGATACAGATCAGCA
TRDN	TCACAGAAGACATAGTGACGACG	TGGCAATAGAGCTTGCTGAAA
CAV3	GACCCCAAGAACATTAACGAGG	GGACAACAGACGGTAGCACC
BIN1	ATGAGGCAAACAAGATCGCAG	CGTGACTTGATGTCGGGGAA
AMP2	TGAGCAGTGCGTCCAGAATTT	CGATCTTGTTTGCCTCATCCC
SCN5A	AGCTGGCTGATGTGATGGTC	CACTTGTGCCTTAGGTTGCC
KIR2.1	GTGCGAACCAACCGCTACA	CCAGCGAATGTCCACACAC
SCN1B	TCCTGCGCTATGAGAATGAGG	TGGTGTTGTGCTCGTAGTTTTC
HCN1	CATGCCACCGCTTTAATCCAG	ATTGTAGCCACCAGTTTCCGA
KCNJ8	GTGATTGCCGTCCGAAATGG	AGTTGGTGAATAGGAACCACCT
KCNH2	CAACCTGGGCGACCAGATAG	GGTGTTGGGAGAGACGTTGC
PPARGC1A	GCTTTCTGGGTGGACTCAAGT	GAGGGCAATCCGTCTTCATCC
NPPA	CAACGCAGACCTGATGGATTT	AGCCCCCGCTTCTTCATTC
NPPB	TGGAAACGTCCGGGTTACAG	CTGATCCGGTCCATCTTCCT
MAPK1	TCTGGAGCAGTATTACGACCC	CTGGCTGGAATCTAGCAGTCT
PRKACA	CAAGGAGACCGGGAACCACTA	CATTCAGGGTGTGTTCGATCTG
MYL2	TGTCCCTACCTTGTCTGTTAGCCA	ATTGGAACATGGCCTCTGGATGGA
MYL7	ACATCATCACCCATGGAGACGAGA	GCAACAGAGTTTATTGAGGTGCCC
HCN4	GGTGTCCATCAACAACATGG	GCCTTGAAGAGCGCGTAG
KCNQ1	CCACCTCAACCTCATGGTG	ACAGTGAGGGCTTCCCAAT
GAPDH	GAGTCAACGGATTTGGTCGT	GACAAGCTTCCCGTTCTCAG

Supplementary Video S1 PSC derived cardiac differentiation process from day –3 to day 12.

Supplementary Video S2 Day 6 CPCs with special texture demonstrated by image streams from day 4 to day 12.

Supplementary Video S3 Differentiation tendency of cells located at the periphery or the center of colonies demonstrated by image streams from day 0 to day 10.

Supplementary Video S4 Beating PSC-CM treated by BI-1347 (0.5 μM, 0–48 h) on day 12.

Supplementary Video S5 LSM 5 live imaging of spontaneous calcium transient in cell clusters. Left: PSC-CMs + BI-1347 group. Right: PSC-CMs – BI-1347 group. Scale bar: 20 μm. Scan speed: 10 Hz.
